# Metagenomic insights into the taxonomic and metabolic diversity of the microbiome of Lake Karum in the Danakil Depression, Ethiopia

**DOI:** 10.1186/s40793-026-00938-z

**Published:** 2026-07-30

**Authors:** Michael C. Macey, Velislava Ilieva, Ben P. Stephens, Benjamin Tatton, Ermias Balcha, Susanne P. Schwenzer, Terry J. McGenity, Hagos Miruts, Felipe Gomez, Barbara Cavalazzi, Karen Olsson-Francis

**Affiliations:** 1 AstrobiologyOU, Earth, Environment and Ecosystem Sciences, The Open University, Milton Keynes, UK; 2 Addis Ababa Science and Technology University, Addis Ababa, Ethiopia; 3 School of Medical Laboratory Science, College of Health Sciences, Hawassa University, Hawassa, Ethiopia; 4 School of Life Sciences, University of Essex, Colchester, UK; 5 Mekelle University, Mekelle, Ethiopia; 6 Centro de Astrobiología, Madrid, Spain; 7 University of Bologna, Bologna, Italy

## Abstract

**Background:**

Hypersaline environments are dynamic ecosystems, the chemistry of which is significantly influenced by climate change, which in turn impacts the microbiota and biogeochemical processes. This study investigates the microbiome of Lake Karum, a hypersaline lake in the Danakil Depression, Ethiopia, with a particular focus on genome-based potential of climate-relevant biogeochemical processes.

**Results:**

The microbiomes of Lake Karum sediments and waters were dominated by halophilic Archaea (*Halobacteriota*) and Bacteria (*Bacteroidota*, *Pseudomonadota*, and *Cyanobacteriota*), with significant variation in community composition among sites, reflecting geochemical heterogeneity. Despite these taxonomic differences, sediment and water metagenomes exhibited broadly overlapping functional gene profiles. Genes involved in denitrification, carbon monoxide oxidation, osmotic stress tolerance, and utilisation of osmolytes were widespread and predominantly affiliated with Halobacteriales, indicating their pivotal role in nitrogen and carbon cycling. Genome‑resolved analyses revealed substantial intrageneric variation in metabolic potential within dominant halobacterial lineages as well as bacterial candidate phyla, including *Candidatus* Bipolaricaulota and *Candidatus* Salsurabacteriota, which encode genes linked to trace-gas metabolism and nitrogen cycling. Notably, a high‑quality metagenome‑assembled genome assigned to the *Candidatus* Salsurabacteriota was recovered that possesses novel combinations of functional genes not previously reported for this lineage.

**Conclusion:**

This study provides a comprehensive genome‑resolved assessment of the taxonomic and functional diversity of the Lake Karum microbiome and identifies microbial taxa with the potential to drive key carbon, nitrogen, and sulfur cycling processes in a hypersaline lake. By revealing previously unrecognised metabolic capabilities within bacterial candidate phyla and highlighting intrageneric functional heterogeneity among dominant halophilic *Archaea*, this work advances understanding of how hypersaline microbial communities contribute to biogeochemical cycling in extreme environments.

**Supplementary Information:**

The online version contains supplementary material available at https://doi.org/10.1186/s40793-026-00938-z.

## Introduction

Surficial hypersaline environments (NaCl > 0.9 M) are cosmopolitan and represent a significant volume of water, particularly in the arid and semi-arid zones that form a third of terrestrial environments [1]. Hypersaline environments are formed by natural and anthropogenic processes [2, 3] and can vary significantly in physicochemical conditions, e.g., salinity, ionic composition, temperature regime and nutrient status [4–9]. There are two main types: thalassohaline, which originate from seawater and typically are circumneutral [1, 8, 10]; and athalassohaline lakes, which have no direct marine influence, and include soda lakes (pH 9–11) that are rich in sodium chloride, sodium carbonate and sodium bicarbonate [1, 11]. These lakes are often surrounded by salt flats formed from water evaporation. Hypersaline environments are dynamic and heavily influenced by climate change and water abstraction, with some hypersaline water bodies being formed [12–15] while others are shrinking and sometimes being converted into desiccated basins [16–20]. A rise in global temperatures and more extensive freshwater abstraction will increase the abundance and the salinity of hypersaline environments [1, 20–22], altering the flux of climate-active volatile compounds to the atmosphere [16–20], and disrupting ecosystem services at both global scales (e.g., the regulation of biogeochemical cycles) and local scales (e.g., salt farming) [13, 23]. As climate-driven salinisation and hydrological change expand the global extent of hypersaline systems and alter their redox regimes, resolving microbially mediated biogeochemical cycles in these environments becomes increasingly critical for understanding potential feedbacks between changing water regimes, atmospheric composition, and ecosystem stability [24–26].

Surficial hypersaline environments host highly specialised microbial communities that sustain active carbon, nitrogen, and sulfur cycling under extreme salinity, supporting both phototrophic and chemoautotrophic primary production [1, 25–27]. Process-based studies of hypersaline environments and cultivation-based studies using halophilic isolates have identified a widespread occurrence of diverse metabolic pathways with direct or indirect climate-relevant effects (e.g., denitrification and carbon monoxide oxidation) [16, 28–35]. Metagenomes generated from the waters and surrounding salts of surficial hypersaline environments have characterised the genetic diversity underpinning these processes [36–44]. However, insight into the functional potential of hypersaline microbiomes can be further enhanced by genome-resolved investigation of variation in pathways linked to trace gas metabolism and biogeochemical cycling.

**Fig. 1:**
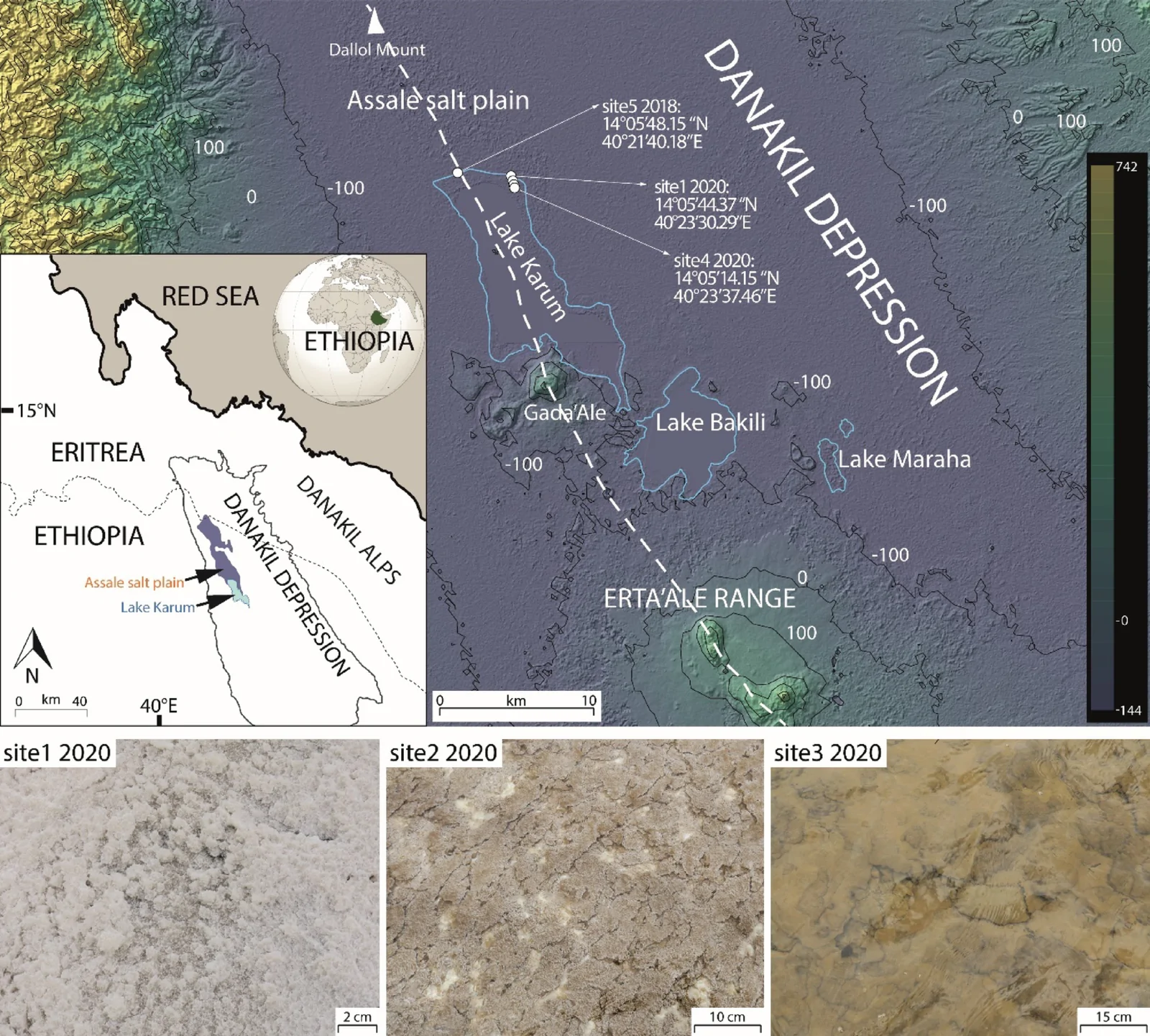
Map and photographs of Lake Karum in the Danakil Depression, Ethiopia. White dots indicate approximate sampling locations. The 2018 sampling site (Site 5) is shown to the northwest of the 2020 sampling transect. The 2020 transect comprises four approximately equidistant sites (Sites 1–4) arranged from shallow to deeper water: Site 1 corresponds to emergent salt crust with no overlying water (0 cm), Sites 2 and 3 represent shallow-water locations (5–10 cm), and Site 4 corresponds to the deepest sampled location (40–50 cm water depth). Elevation relative to sea level (m) is indicated

In this study, Lake Karum (also known as Lake Assale, and alternatively spelled as Asale, Asshale or Assele, As’Ale), an Ethiopian hypersaline lake [45], was selected for metagenomic characterisation to study the taxonomic and functional diversity of its microbiome. Lake Karum is located in the Danakil Depression (Fig. 1), a region in the northern part of the geologically active Afar Depression of the East African Rift System [6]. The Danakil Depression NNW-SSE-oriented basin is occupied at the surface by the wide Assale salt plain, which also includes the Dallol volcano and the Erta’Ale volcanic range (Fig. 1). This area is in an active rift zone with frequent earthquakes, volcanic activity and is one of the geographically lowest areas in Africa. Periodic marine ingressions during the Pleistocene resulted in thick evaporite deposits that drove the formation of recent salt deposits of the Assale salt plain [46, 47]. Freshwater rivers enter the plain through underground pathways, dissolving pre-existing salt deposits [46, 47]. Nowadays, Lake Karum is an elongated, shallow, ephemeral, and highly saline lake that periodically floods the salt plain due to rainfall. Future perturbations in freshwater input due to climate change would greatly impact the lake and its microbiome.

The microbiomes of many hypersaline environments have been well studied in terms of their taxonomic diversity [2, 4, 48–50]. Previous studies of Lake Karum [7, 51, 52] demonstrated that the microbial community was dominated by halophilic *Archaea* and *Bacteria* and was stable, with similar microbial diversity observed in samples collected over multiple years [51]. Dominant archaeal taxa were shown to be* Halobacteriota* and *Candidatus* Nanohaloarchaeaota, and abundant bacterial taxa were *Salinibacter*, *Cyanobacteriota*, *Candidatus* Bipolaricaulota and *Candidatus* Patescibacteria [7, 51, 52]. Taxonomic studies are instrumental in deciphering the diversity, but do not identify the functional diversity in the microbial community [53] and there may be significant variation owing to intra-species and intra-genera variation in functional potential [54–57]. Genome‑resolved metagenomics offers a critical means of identifying variation in functional potential and diversity that cannot be inferred from taxonomic profiles alone. In particular, it enables assessment of the extent to which closely related halophilic *Archaea* and bacterial candidate phyla differentially contribute to trace gas metabolism and biogeochemical cycling. Building upon metagenomic studies of hypersaline environments that have characterised a range of climate-relevant metabolic pathways [34, 49, 58, 59], this study applies geochemistry, taxonomy and genome‑resolved metagenomics to characterise the microbiome of Lake Karum.

## Material and methods

### Sampling site and sample collection

Water and sediment samples were collected from the margins of Lake Karum during two field campaigns, in January 2018 and February 2020. In January 2018, samples (n = 3) were collected from a single site located at 14°05ʹ48.15ʺ N, 40°21ʹ40.18ʺ E, hereafter referred to as Site 5 (Fig. 1), which is spatially distinct from the 2020 sampling transect and was used for comparative analysis. During February 2020, samples were collected from four approximately equidistant sites (Sites 1–4) along a transect extending between 14°05ʹ44.37ʺ N, 40°23ʹ30.29ʺ E and 14°05ʹ14.15ʺ N, 40°23ʹ37.46ʺ E (Fig. 1). These sites represent a gradient in water depth, with Site 1 located on emergent salt crust with no standing water (0 cm), Sites 2 and 3 located in shallow water (5–10 cm), and Site 4 representing the deepest sampled location (40–50 cm). Sediments were collected at two distinct transect points per site (n = 8). Sediment samples were collected from Sites 1–4 using a sterile spatula for each site and stored in a sterile Nalgene bottle. The spatula was sterilised with 70% IPA Azo wipes between sampling.

Water samples were collected from Sites 2–4 at two distinct transect points (n = 6). Water samples were collected aseptically using sterile 50 ml plastic syringes. For geochemical analysis, water samples were stored in sterilised 250 ml polypropylene bottles (Nalgene). For DNA extraction and characterisation of the microbiome, 500 ml of water was passed through a 0.22 μm filter (Starlabs) in duplicate for each site; no water was present at Site 1. The samples were stored at 4 °C before being shipped to The Open University within seven days of sampling. On arrival in the laboratory, the samples were sub-sampled and stored at −20°C for DNA extraction (0.22 µm syringe filters and 30 g of sediment) and at ambient conditions (approximately 100 ml of water and 10 g of sediment) for further geochemical characterisation.

### Geochemical analysis

Temperature (uncertainty of 0.5°C) and pH (uncertainty of 0.01 pH units) were measured in situ using a Mettler Toledo FiveGo probe. The water activity of the samples (the free water available for chemical and microbial processes) was measured in the laboratory using a Novasina LabMaster A_W_ meter at 36°C, applying the manufacturer’s instructions. The water activity meter was calibrated using saturated salt solutions (magnesium nitrate and chloride, potassium acetate, chloride and sulfate, lithium bromide and chloride, sodium bromide and chloride, and caesium fluoride). The total composition of dissolved elements was measured using Inductively Coupled Plasma Atomic Emission Spectrometry (ICP-OES; Agilent model 5110), as previously described [60]. Salts (5 g) were dissolved in MilliQ water to a final volume of 15 ml and processed in parallel with the water samples. Principal component analysis (PCA) was performed on the geochemical data with the program ClustVis [61] to explore patterns in elemental composition among samples. Geochemical variables were centred by subtracting the column means and scaled to unit variance prior to analysis. PCA was calculated using singular value decomposition with imputation, and ordination plots were used to visualise multivariate geochemical relationships among samples.

### DNA extraction

For the 2018 samples, DNA was extracted from each sample in triplicate, generating three technical replicates per sample whilst for the 2020 sample two technical replicates were extracted. DNA was extracted using the modified XS buffer extraction technique in a clean hood (PURAIR, Air Science) [60, 62]. All reagents were UV sterilised, except for the potassium ethyl xanthogenate. After all of the XS buffer reagents were combined, the XS buffer was filter–sterilised through a 0.22 μm filter. Samples were extracted and purified, as previously described [63]. In detail, 10 g of sediment was suspended in 30 ml of PCR-grade molecular water (Sigma-Aldrich®) and vortexed for 20 min. The sediment was pelleted by centrifugation at 1000 × *g*, for 5 min, and the supernatant was centrifuged for 5 min at 4700 × *g* through a 30 kDa UV sterilised filter (Merck®). This filter retained all DNA > 800 bp, focusing DNA extraction on longer fragments of DNA and removing smaller DNA fragments, RNA, proteins, and other cellular debris [57]. For the water, the filters were extracted from the plastic casing, suspended in 10 ml of PCR-grade molecular water (Sigma-Aldrich®) and vortexed for 20 min. To further dilute the salts from the filters, 10 ml was combined with 30 ml of PCR-grade molecular water (Sigma-Aldrich®) and centrifuged through a 30 kDa UV sterilised filter (Merck®), as above. All filters were washed with 400 µl of 1 M Tris HCl (pH 8), which was then collected and combined with 400 µl of XS buffer [62]. DNA was extracted using the XS buffer DNA extraction technique, with the addition of a freeze–thaw step, during which the samples were incubated at 65°C, transferred to −80°C, and then incubated again at 65°C, with each step lasting 30 min [57]. Nucleic acids were precipitated with one volume of ethanol and 4 µl of GlycoBlue Coprecipitant (ThermoFisher®), as per the manufacturer’s instructions. The supernatant was discarded, and the pellet was washed in 200 µl of 70% (v/v) ice-cold ethanol and air dried. To remove excess salts, the nucleic acids were re-suspended in 1.5 ml of PCR-grade water and centrifuged at 12,000 ×*g* for 5 min through a 500 µl volume UV-sterilised 30 kDA filter (Merck®). The filter was inverted and transferred to a new tube, and 40 µl of PCR-grade water was added to the filter and centrifuged again at 12,000 × *g* for 5 min. DNA was quantified using the high-sensitivity DNA assay for Qubit fluorometric quantitation kit, as per manufacturer’s instructions (ThermoFisher®). For each stage of the extraction process, nuclease-free water (Sigma-Aldrich®) was processed in parallel as an additional negative control.

### 16S rRNA gene PCR amplification and sequencing

Partial 16S rRNA genes were amplified and sequenced from 28 samples and three negative controls. With regard to the 2018 field campaign, three technical replicates of a sediment sample were included to assess sample heterogeneity. For the 2020 samples, amplicons were generated from each of the water samples collected at Sites 2–4 (six amplicons total) and duplicate technical replicates from each of the sediment samples collected from Sites 1–4 at two distinct transect points (sixteen amplicons total). The three negative controls that were also sequenced included one negative control extracted in parallel with the 2018 samples and two from the 2020 samples. The V4-V5 region of the microbial 16S rRNA gene was amplified using the bacterial primers com1 and com2 [64, 65]. The PCR mixture contained (per 25 µl): 1 × PCRBIO Ultra Polymerase red mix (PCR BIOSYSTEMS, United Kingdom), 0.4 μM forward primer and 0.4 μM reverse primer. DNA samples for the PCR mixture were diluted to a maximum of 1 ng/μl. PCR products were purified with calibrated Ampure XP beads (Agencourt Bioscience, USA). A DNA library was created following the Illumina TruSeq protocol, and 16S rRNA gene sequencing was performed using v.3 chemistry (2 × 250 bp) on the Illumina MiSeq platform by Molecular Research LP (Texas, USA). The raw sequence reads generated by the amplicon sequencing were processed in R using DADA2 (v1.36.0) for trimming, filtering, denoising, merging and taxonomic classification [66]. The forward and reverse reads were trimmed to 260 bp and 150 bp based on quality scores, respectively. Sequences containing ambiguous bases were discarded, and the expected error rate was set to 1. Dereplication, merging of forward and reverse reads, and removal of chimeric sequences, were done with default parameters. Taxonomic assignment of the resulting amplicon sequence variants (ASVs) was performed with the SILVA database, v138.2 [67]. For phylogenetic analyses, ASVs were aligned using the DECIPHER package (v2.26) [68], and a maximum-likelihood tree was constructed with the phangorn package (v2.11.1) [69]. Phylogenetic distance between ASVs was determined with the *cophenetic()* function. The microbial richness and diversity of the obtained samples were assessed using the *plot_richness()* function of the phyloseq package (v1.42.0) [70] and default parameters. All amplicons were normalised by applying a variance stabilizing transformation with the *varianceStabilizingTransformation()* function of the DESeq2 package (v1.38.3) with default parameters. Permutational multivariate analysis of variance (PERMANOVA) was performed in RStudio 4.0.2 using the vegan package (v2.7–2) [71–73] with 999 permutations to assess the differences in microbial community composition among sample types. To identify the specific taxa driving the differences between water and sediment communities, a Similarity Percentages (SIMPER) analysis was performed using the vegan package (v2.7–2) [71–73]. Microbial community composition was visualised using non-metric multidimensional scaling (NMDS) based on Bray–Curtis dissimilarities. The non‑metric multidimensional scaling (NMDS) plot was created with the *ordinate()* function from the phyloseq package (v1.42.0) with *distance* = *”bray”*. Geochemical variables were fitted onto the NMDS plot with the *envfit()* function from the vegan package (v2.7–2) with default parameters. A Pearson correlation plot was created with the corrplot package (v0.95) [74] and default parameters*.* The geochemical data was centred by subtracting column means and scaled by dividing the centred data by the standard deviation prior to analysis.

### Metagenome sequencing and analysis

Metagenomics was carried out on the 2020 samples. To ensure sufficient DNA for sequencing, DNA was pooled from biological replicates collected at each transect point (i.e., sediment from within each site (S1-S4) and water from all 2020 samples (W)). Sequencing was performed using paired-end sequencing (2 × 150 bp) on an Illumina HiSeq 4000 (IGA Technology Services, Italy). Short sequences and sequences of poor quality were excluded using the program Trimmomatic (v0.36) (default settings) [75], and the quality of the metagenomes was assessed using QUAST, including the MetaQUAST expansion (v5.0.0) (Supplementary Table 1) [76]. Reads were assembled using MEGAHIT (v1.1.2) (default settings) [77] and annotated using Prokka (v1.15.4) (default settings) [78]. The taxonomic profile of the metagenomes was analysed using Kaiju [79]. The presence of functional genes involved in biogeochemical cycling was assessed using DRAM (v0.1.2) with default parameters [80].

The Trimmomatic, Megahit, Prokka and DRAM steps were performed using Kbase [81]. DRAM uses two annotation databases, KEGG Orthology and Pfam, thus generating two annotations for each hit. The KEGG Orthology database assigns a K number, which represents a functional ortholog, to each annotated gene. Unclassified reads were removed prior to analysis, and the abundance of the classified reads was normalised by applying variance stabilising transformation with the *varianceStabilizingTransformation()* function of the DESeq2 package (v1.38.3) with default parameters. Functional genes involved in the metabolism of climate-relevant compounds were identified in the metagenome using Blast + [82] and verified by comparison to the Uniprot database [83, 84].

### Binning of metagenome sequences

Contigs were binned into metagenome-assembled genomes (MAGs) using MaxBin2 (v2.0) [85], Metabat2 (v1.7) [86] and CONCOCT (v1.1) [87]. The MAGs were then processed into a non-redundant set of MAGs using the programs DasTool [88] and DRep [89] with default settings. The completeness, contamination and heterogeneity of these MAGs were assessed using the program CheckM (1.0.13) [90] and refined using VizBin [91]. The taxonomy of the MAGs was assessed using GTDB-Tk [92–94]. The presence of specific functional genes was screened for using BlastKOALA [82], DRAM [80], and Prokka [78]. Protein‑coding genes predicted from the metagenomes were annotated using Prokka, with functional identities verified against curated reference databases (KEGG via GhostKOALA and UniProt). For each functional gene of interest, taxonomic affiliation was initially inferred from the best BLAST match against the UniProt database, based on amino acid sequence similarity and alignment quality. Where functional genes were located on contigs assigned to metagenome‑assembled genomes (MAGs), taxonomic identity was assigned based on the MAG‑level classification, as determined using GTDB‑Tk.

## Results and discussion

Lake Karum, a hypersaline lake in the Danakil Depression in Ethiopia, was studied using a DNA-sequencing and geochemistry-based approach. The water surface temperature at each site was between 28 and 30°C with a pH between 6.7 and 8.0, and water activity between 0.746 and 0.760. The water samples contained high amounts of sodium (92,500–137,000 mg kg^−1^), calcium (20,100–25,700 mg kg^−1^), magnesium (3880–4500 mg kg^−1^) and sulfur (159–306 mg kg^−1^) (Table 1 and Supplementary Table 2). Trends in intersample variability (e.g., lower Na, S and P and higher K at Site 3 and Site 4 compared to Site 1 and 2) were potentially due to the thickness of the salt crust and distance from the lake water, in addition to possible variation in evaporative processes. PCA of the geochemical dataset showed grouping by both sample type (sediment and water) and sampling year (2018 and 2020) (Supplementary Fig. 1), indicating underlying differences in elemental composition among samples. Differences in chemistry between samples can possibly be attributed to spatial variation and annual variation in meteoric water inputs.

**Table 1:** The abundant elements generated from Lake Karum Water and Sediment samples (mg kg^−1^). Numbers are shown to three significant figures. < LoD represents below the limit of detection

	Sediment					Water			
Site	1	2	3	4	5	2*	3	4	5
Year	2020	2020	2020	2020	2018	2020	2020	2020	2018
Na	274,000	29,300	9890	5830	255,000	137,000	125,000	111,000	92,500
Ca	4480	8150	5100	4920	3840	25,700	25,100	22,400	20,100
K	89.4	84.2	< LoD	34.5	85.0	4870	4640	4110	6050
Mg	604	618	370	231	664	4500	4350	3880	4150
S	869	3550	2250	2700	307	165	167	159	306
P	226	244	156	99.4	< LoD	29.1	21.5	< LoD	< LoD
Se	328	29.8	77.6	136	< LoD	40.2	67.8	75.4	< LoD
Sr	67.7	89.2	56	40.5	68.6	432	418	382	392

*No water was present at Site 1

< LoD represents below Limit of Detection

### Characterisation of microbial communities

#### Taxonomic profiles of Lake Karum and the other surficial hypersaline environments

Thirty one 16S rRNA gene amplicon libraries were generated and sequenced - six from 2018 (Site 5, with triplicate technical replicates), 22 from 2020 (biological duplicate water samples from Sites 2–4 and sediment samples from two transect points per site from Sites 1–4, with duplicate technical replicates of each) and three negative controls (one negative control extracted in parallel with the 2018 samples and two from the 2020 samples) (Supplementary Table 3). A total of 1,121,042 sequences were obtained after quality control (Supplementary Table 4). Diversity indices (Shannon, Simpson, and Inverse Simpson) indicated no significant difference between the two years (Supplementary Table 5).

**Fig. 2:**
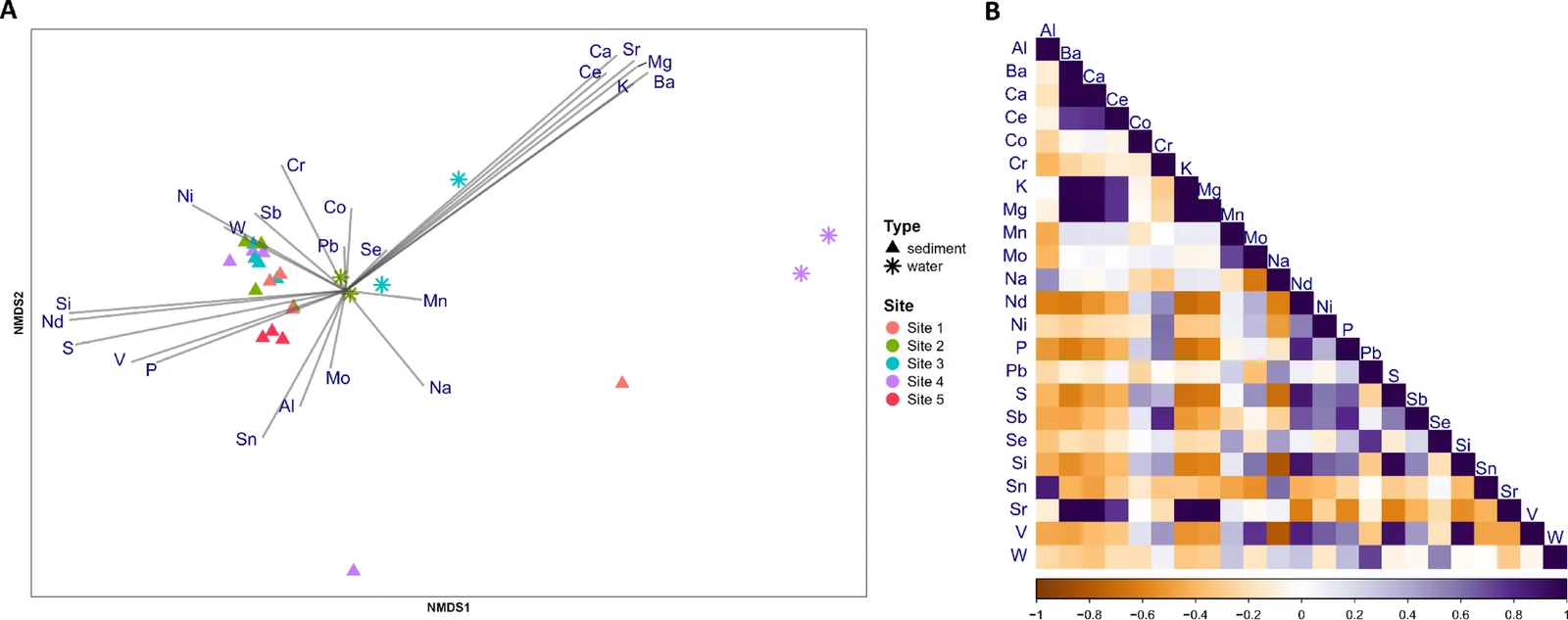
A Non‑metric multidimensional scaling (NMDS) of microbial community composition based on Bray–Curtis dissimilarities of 16S rRNA gene amplicon data from Lake Karum sediments and waters. Points represent individual samples and are coloured by sampling site (Sites 1–5; see Table 1) and shaped by sample type (sediment or water). Vectors indicate the direction and relative strength of correlations between community composition and scaled geochemical variables, with only variables included in the ordination shown. B Pearson correlation of geochemical variables (− 1-—a perfect inverse relationship; 0-—no relationship; 1-—a perfect linear relationship)

PERMANOVA analyses based on Bray–Curtis dissimilarities indicated that microbial community composition differed significantly among sampling sites (Sites 1–5; *p* = 0.001, *R*^2^ = 0.286) and between sample types (water versus sediment; *p* = 0.002, *R*^2^ = 0.153), with homogeneity of multivariate dispersion confirmed for both comparisons. In contrast, differences between sampling years (2018 versus 2020) could not be robustly assessed using Bray–Curtis distances due to significant heterogeneity of dispersion between groups. Unconstrained analyses identified a suite of co-varying geochemical parameters, with vectors for elements such as barium, aluminium and neodymium, as well as major ions including sodium, magnesium and calcium, showing strong correlations with sample distribution in ordination space, collectively explaining 67.7% of the observed variance (Fig. 2). Phylogenetic beta-diversity based on weighted UniFrac distances also showed a significant separation between water and sediment communities (*p* = 0.021, *R*^2^ = 0.222), whereas no significant effect of sampling year was detected. The sediment communities were associated with mainly geochemical parameters that present at low abundance, which had positive Pearson correlation (*r* > 0) between each other (e.g., Ni, W, Si, Nd, P). The site 3 water communities were associated with major elements that also had positive Pearson correlation (e.g., Ca, Mg, K, Sr), as well as Ce. Collectively, these results indicate that microbial communities are shaped by complex, multivariate geochemical structuring rather than responses to a single environmental gradient, with unmeasured physicochemical parameters likely contributing to these distributions.

**Fig. 3:**
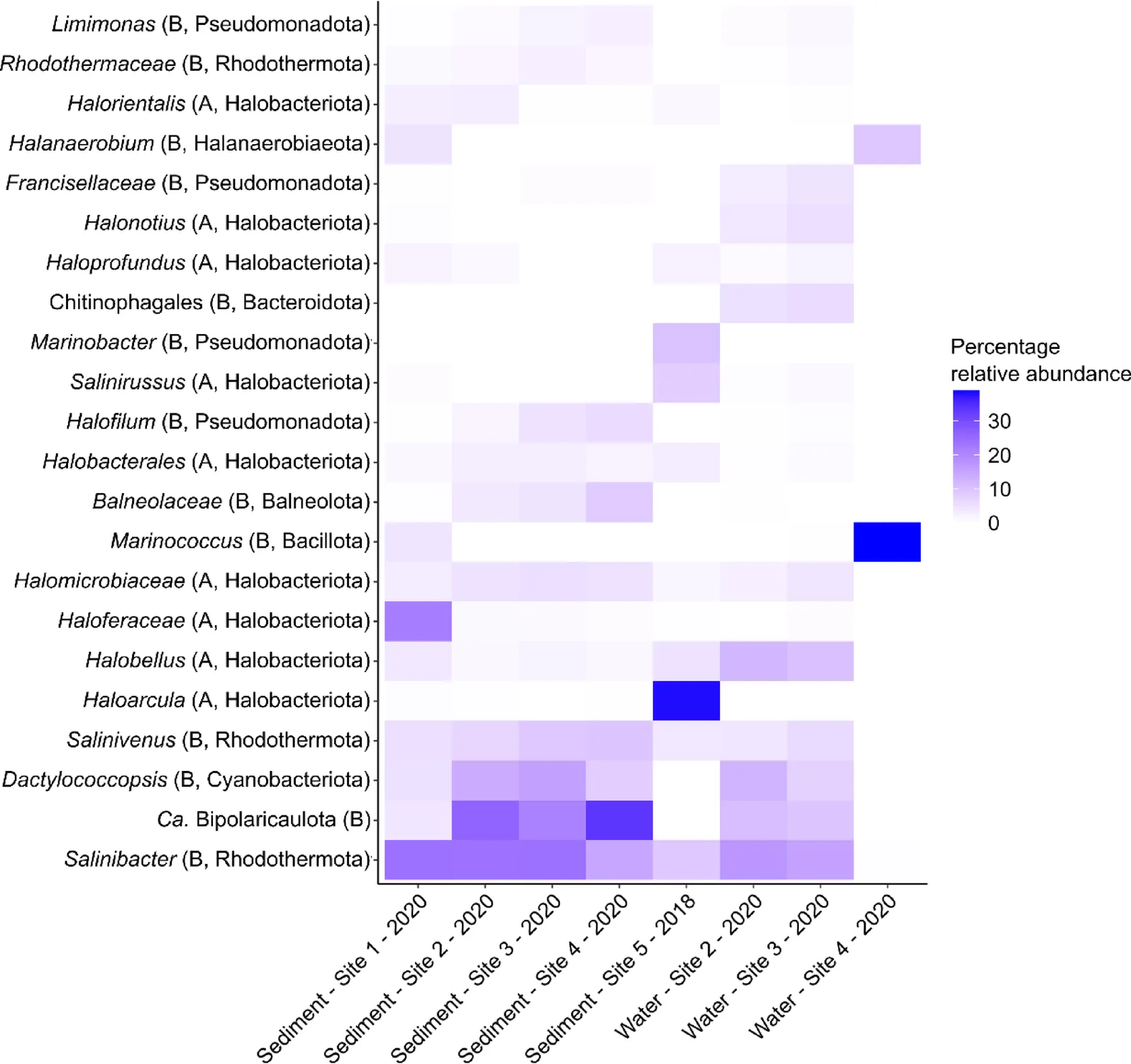
Pooled 16S rRNA gene amplicon community profiles of Lake Karum sediment and water are shown at the genus level (> 2% relative abundance). Taxa are ordered from bottom to top by total read abundance

Taxonomic assignment demonstrated that the majority of sequences were affiliated with the phyla *Halobacteriota* (e.g. *Haloarcula, Halobellus,* and *Halomicrobiaceae*), *Bacteroidota* (*Salinibacter* and *Salinivenus*), *Candidatus* Bipolaricaulota, *Cyanobacteriota* (*Dactylococcopsis*), *Bacillota* (e.g., *Marinococcus*), and *Pseudomonadota* (e.g. *Marinobacter* and *Limimonas*) (Fig. 3). While these taxa were detected across all samples, their relative abundances varied significantly between water and sediment samples, consistent with patterns observed in beta-diversity analyses (Fig. 3). Sediment communities were consistently dominated by *Salinibacter*, *Salinivenus*, *Haloarcula,* and *Candidatus* Bipolaricaulota. Water communities were characterised by high relative abundances of *Marinococcus* and *Halobellus*, alongside genera that were rare or sporadically detected in sediments, including *Halonotius*. The marked differentiation of the Site 4 water community from other samples was largely attributable to the dominance of *Marinococcus*. SIMPER analysis identified *Candidatus* Bipolaricaulota and *Salinibacter* as the primary contributors to dissimilarity between the different sites and between water and sediment, contributing 11.2% and 9.6% respectively to the observed variance. These taxonomic patterns align with the clear separation of samples by habitat type identified by PERMANOVA and NMDS ordination (Fig. 2).

Further taxonomic analysis of the sequences originally classified as chloroplasts revealed that these reads were from chloroplasts of unicellular Chlorophyta from the genera *Crustomastix* and *Prasiola*. Oxygenic photoautotrophs identified in the 16S rRNA gene dataset comprised both Cyanobacteriota and eukaryotic algae [95, 96]. Chloroplast sequence analysis indicated that the eukaryotic phototroph community was dominated by taxa closely related to *Crustomastix*, which has previously been detected in halite environments [97], alongside *Prasiola* spp., which are known to tolerate fluctuating salinity [98]. *Cyanobacteriota* are typically dominant primary producers in hypersaline microbial mats and crusts, where community structure is often shaped by a limited number of highly halotolerant taxa [99]. In contrast, hypersaline water columns are frequently dominated by the chlorophyte *Dunaliella,* but no *Dunaliella* sequences were detected in the amplicon dataset. The predominance of *Cyanobacteriota*, including *Dactylococcopsis*, together with chloroplast sequences affiliated with halotolerant algae, is consistent with observations from other saline and stratified environments [100].

The 16S rRNA gene amplicon profiles generated in this study are largely consistent with those from previous studies of Lake Karum [51, 52], and nearby ephemeral pools in the Danakil depression [52]. In these studies [51, 52] profiles were dominated by *Halobacteriota, Candidatus* Nanohaloarchaeota*, Pseudomonadota, Bacteroidota,* and *Candidatus* Bipolaricaulota; the absence of sequences classified as *Candidatus* Nanohaloarchaeota by amplicon sequencing in this study when they were detected in prior studies [51, 52] is likely a consequence of primer selection, as we detected them in metagenomes (see later). All ASVs that were found in the negative controls were removed from both Lake Karum samples. The three negative controls produced 15,000–21,000 reads and differed in dominant taxa. The 2018 control (K1.NRT) was mainly *Stenotrophomonas* (45.17%), *Delftia* (27.13%) and *Achromobacter* (26.41%). Both 2020 controls showed different profiles and were dominated by either *Ammonifex* (58.39%), or *Psychrobacter* (44.16%), *Anaerococcus* (16.52%) and *Achromobacter* (11.67%) (Supplementary Table 3).

For the metagenome sequencing, the biological and technical replicates from the 2020 campaign (Fig. 1) were pooled to ensure sufficient DNA for sequencing. This resulted in five metagenomes: four from sediment (one per site across sites 1–4) and one from water (one per site across sites 2–4) (Supplementary Fig. 1). The archaeal phylum *Halobacteriota* dominated these metagenomes, accounting for 60–88% of the sequences. Within this phylum, 42 genera were identified, led by the particularly abundant* Haloplanus* and *Halobaculum*. The most abundant bacterial phyla were Bacteroidota (9–26%) (*Salinibacter*) and *Pseudomonadota* (1–9%) (*Thioalkalivibrio, Halomonas, Marinobacter*). There is variability between sediment samples, supporting the variation identified by the 16S rRNA gene sequencing. *Cyanobacteriota*, *Bacillota* and *Actinomycetota* were also detected but at < 2% relative abundance. Taxonomic profiling of the metagenomes also identified members of candidate phyla that were not detected in the 16S rRNA gene profiles, specifically *Candidatus* Nanohaloarchaeaota (1–1.5% relative abundance). *Candidatus* Nanohaloarchaeaota represents a candidate phylum of *archaea* within the DPANN that has been shown to be present in Lake Karum [51, 52, 59] and other diverse hypersaline ecosystems [10, 101].

### Functional profiles of surficial hypersaline environments

**Fig. 4:**
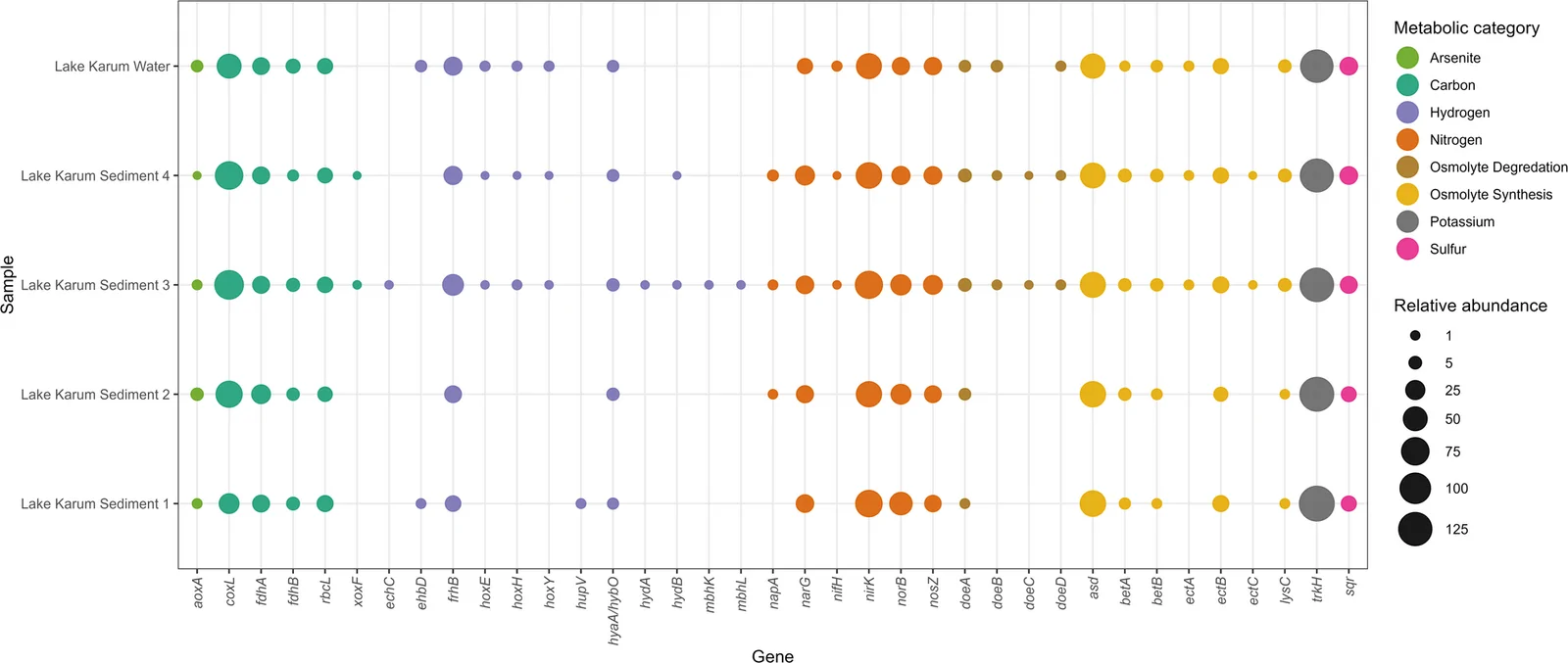
Bubble plot showing the relative abundance of selected functional marker genes across Lake Karum sediment (Sediments 1–4) and water metagenomes. Bubble size represents variance‑stabilised gene abundance values derived from DESeq2 normalisation, allowing comparison across samples. Colours indicate metabolic functions (carbon, hydrogen, nitrogen, osmolyte degradation, osmolyte synthesis, potassium, sulfur, and arsenite). Bubble sizes are scaled continuously according to normalised abundance values (variance‑stabilising transformation). The size scale shown in the legend corresponds to representative transformed values rather than absolute read counts. Carbon metabolism: *rbcL* (ribulose-1,5-bisphosphate carboxylase/oxygenase large subunit), *coxL* (carbon monoxide dehydrogenase large subunit), *fdhA* and *fdhB* (formate dehydrogenase subunits), and *xoxF* (methanol dehydrogenase). Nitrogen cycle: *nifH* (nitrogenase iron protein), *napA* (periplasmic nitrate reductase), *narG* (respiratory nitrate reductase), *nirK* (copper-containing nitrite reductase), *norB* (nitric oxide reductase), and *nosZ* (nitrous oxide reductase). Hydrogen metabolism: *hoxE*, *hoxH*, and *hoxY* (bidirectional hydrogenase subunits), *hyaA/hybO* and *mbhL* ([NiFe] hydrogenase large subunits), *mbhK* and *hydB* (associated electron transfer subunits), *hydA* ([FeFe] hydrogenase catalytic subunit), *ehbD* and *echC* (energy-converting hydrogenase subunits), *hupV* (hydrogen uptake protein), and *frhB* (coenzyme F420-reducing hydrogenase subunit). Sulfur metabolism: *sqr* (sulfide:quinone oxidoreductase). Arsenic metabolism: *aoxA* (arsenite oxidase). Osmolyte synthesis: *betA* (choline dehydrogenase), *betB* (betaine aldehyde dehydrogenase), *lysC* (aspartokinase), *asd* (aspartate-semialdehyde dehydrogenase), *ectB* (diaminobutyrate aminotransferase), *ectA* (diaminobutyrate acetyltransferase), and *ectC* (ectoine synthase). Osmolyte degradation: *doeA* (ectoine hydrolase), *doeB* (N-acetyl-diaminobutyrate deacetylase), *doeD* (aspartate-semialdehyde dehydrogenase in the degradation pathway), and *doeC* (diaminobutyrate transaminase). Ion transport: *trkH* (potassium uptake protein)

Functional gene analyses were used to assess the distribution of metabolic potential across Lake Karum sediment and water communities, and interpretations are framed in terms of genomic potential. Reflecting their dominance of the metagenomes, most of the genes detected in the Lake Karum datasets were assigned to the order *Halobacteriales* at the maximum level of taxonomic resolution, with *Bacteroidota*, *Pseudomonadota, Cyanobacteriota* and candidate taxa detected in specific instances. In spite of geochemical differences between sediment and water samples, the metagenomes exhibited broadly similar functional gene profiles across carbon, nitrogen, hydrogen and osmolyte-related pathways. Differences between environments were limited to a small number of functions with restricted distributions. Genes encoding a lanthanide-dependent methanol dehydrogenase (*xoxF*) and periplasmic nitrate reductase large subunit (*napA*) were detected at low abundance in a subset of sediment metagenomes but were absent from water samples. These patterns indicate largely overlapping functional potential between sediment and water communities, with limited environment-specific differentiation at the level of gene presence and relative abundance (Fig. 4).

Carbon and nitrogen fixation are key processes that drive primary production and sustain the productivity of an ecosystem [5]. Genes encoding enzymes involved in carbon and nitrogen fixation were detected across the Lake Karum metagenomes. Genes encoding the large subunit of ribulose-1,5-bisphosphate carboxylase/oxygenase (*rbcL*), indicative of carbon fixation potential, were consistently detected in all sediment and water metagenomes at comparable relative abundances. In contrast, genes encoding nitrogenase (*nifH*) were detected only at low abundance and in two sediment metagenomes and the water metagenome, indicating a limited and spatially variable potential for biological nitrogen fixation. Both the *rbcL* and *nifH* sequences were predominantly affiliated with *Cyanobacteriota*, consistent with other hypersaline environments [50, 99, 102], such as salt crusts in the Atacama Desert, the Makgadikgadi Pan in Botswana [5], and Lake Diamante in Argentina [103]. Taxonomic assignments of these sequences further resolved to the cyanobacterial genera *Dactylococcopsis* and *Halothece,* both of which contain halophilic and halotolerant strains and have been detected in diverse hypersaline environments [104–108]. These assignments refine the 16S rRNA gene observations by indicating that members of *Dactylococcopsis* and *Halothece* may also represent important contributors to oxygenic primary production within Lake Karum.

Genes associated with sulfur metabolism were dominated by those encoding sulfide oxidation rather than sulfate reduction, with the sulfide:quinone oxidoreductase gene (*sqr*) detected across all sediment and water metagenomes. The presence of sulfur-oxidation potential across samples did not show a clear correspondence with measured sulfur concentrations, suggesting that sulfur cycling (including cryptic cycling) capacity is broadly distributed within the Lake Karum microbial community.

With regard to dissimilatory nitrogen metabolisms, genes encoding the membrane-bound nitrate reductase (*narG*) were detected in all Lake Karum sediment metagenomes and in the water metagenome, indicating widespread potential for nitrate reduction. Genes encoding the periplasmic nitrate reductase large subunit (*napA*) were detected only in sediment metagenomes, suggesting that this pathway may be favoured in sedimentary communities. Downstream components of the denitrification pathway, including nitrite reductase (*nirK*), nitric oxide reductase (*norB*), and nitrous oxide reductase (*nosZ*), were detected across both the sediment and water metagenomes, although their relative abundances varied among samples. Where taxonomic assignment was possible, these genes were predominantly affiliated with halobacterial taxa, with additional contributions from *Salinibacter* and bacterial candidate phyla such as *Candidatus* Bipolaricaulota. Denitrification is a common energy-generating metabolism in hypersaline environments that is widespread within *Halobacteriota* [16, 31, 42], although it can be strongly inhibited by increasing salinity [20]. These genes have also previously been detected in *Salinibacter* genomes [109]. Together, these results indicate that the genetic potential for partial and complete denitrification is broadly distributed within the Lake Karum microbiome, consistent with observations from other hypersaline environments.

Screening for genes relating to the dissimilatory metabolism of trace gases was performed because of the noted potential for trace gas metabolism in hypersaline environments [110–112]. Screening identified genes for carbon monoxide oxidation and hydrogen metabolism across the Lake Karum metagenomes. Genes encoding the large subunit of the aerobic carbon monoxide dehydrogenase (*coxL*) were detected in all sediment and water metagenomes and represented one of the most abundant carbon-cycling associated genes in the dataset. Normalised abundances of *coxL* were broadly comparable to those of core glycolytic genes across the metagenomes, indicating that the potential for carbon monoxide oxidation is of a similar order of magnitude to central carbohydrate metabolism within the community. Where taxonomic assignment was possible, *coxL* sequences were affiliated predominantly with halobacterial taxa, and a minority were also detected in bacterial lineages including *Limimonas* and *Candidatus* Salsurabacteriota and *Candidatus* Bipolaricaulota. In addition, a range of hydrogenase-encoding genes (including [NiFe] hydrogenase, large (catalytic) subunit-*hyaA/hybO,* [NiFe] hydrogenase, large subunit-*hoxH,* [FeFe] hydrogenase, catalytic subunit-*hydA,* and uptake [NiFe] hydrogenase large subunit- *hupV*) was detected across samples, although their distributions were variable between the Lake Karum metagenomes. To date, all aerobic or aerotolerant carbon monoxide (CO)-oxidising bacteria use the *coxMSL-*encoded enzyme to oxidise CO to carbon dioxide (CO_2_) [113–115]. This enzyme is used by carboxydovores, which oxidise CO as an energy source, and carboxydotrophs, which can utilise CO as both an energy and a carbon source [116, 117]. A high abundance of carbon monoxide dehydrogenase-encoding genes was previously reported in metagenomes from Lake Karum and other hypersaline environments in the Danakil depression [59]. One factor that may contribute to the prevalence of this genetic potential in Lake Karum, further to the atmospheric supply of relevant compounds, is the ground seepage of gases such as CO_2_, CO, N-species, and S-species from the active Afar rift on which this lake is located [118, 119]. However, carbon monoxide dehydrogenase encoding genes are present [11, 44, 120–125], actively expressed [27, 34], and at high relative abundance [40, 58, 121, 125] across multiple hypersaline environments. This consistent detection indicates that CO-oxidation might be a potential cosmopolitan metabolism within hypersaline environments.

Metabolisms associated with the oxidation of small organic compounds were assessed to evaluate their potential as energy sources, with formate specifically highlighted as an electron donor for multiple archaeal species [39, 49, 126]. Genes encoding formate dehydrogenase (*fdhA* and *fdhB*), which catalyse the oxidation of formate to carbon dioxide, were detected across all Lake Karum sediment and water metagenomes, indicating a broadly distributed potential for formate utilisation. In contrast, genes encoding the lanthanide-dependent methanol dehydrogenase (*xoxF*), also proposed to be capable of formaldehyde oxidation [127], were detected only at low abundance in two sediment metagenomes and were absent from water samples. This detection of *xoxF* is consistent with methanol oxidation potential being limited and spatially restricted within the system. *xoxF* sequences clustered outside the characterised diversity of this gene [128] and only showed a maximum of 80% sequence identity with reference sequences, showing the highest similarity to MAGs of the bacterial lineage *Candidatus* Salsurabacteriota generated in this study. However, lanthanides that confer activity to the enzyme (Neodymium and Cerium) were shown to be present in the Lake Karum samples at concentrations that would induce constitutive expression of this gene (> 50 nM) [129] and this detection in hypersaline environments provides deeper insights into the possible role of this gene in extreme environments. These results indicate that while formate oxidation may represent a common metabolic capability in the Lake Karum microbiome, the genetic potential for methanol oxidation is restricted to the sediments.

Genes associated with osmotic stress tolerance and compatible solute metabolism were widespread across the Lake Karum metagenomes, reflecting adaptation to hypersaline conditions. The most consistently detected osmotic stress response was potassium uptake, with the potassium transporter gene *trkH* present at high relative abundance across the Lake Karum metagenomes, consistent with rapid intracellular potassium accumulation as an initial response to osmotic upshift and maintenance of cellular turgor under hypersaline conditions [130, 131]. Genes encoding the biosynthesis pathway for ectoine, a well-characterised compatible solute system in halophilic microorganisms, were also detected in the Lake Karum metagenomes, with partial detection of downstream genes (*L*-2,4-diaminobutyrate acetyltransferase *ectA*, *L*-2,4-diaminobutyric acid transaminase *ectB*, and *L*-ectoine synthase *ectC*) in a subset of sediment metagenomes. Genes encoding enzymes involved in ectoine degradation (*doeA-D*) were also present, indicating turnover of this osmolyte within the community.

Taxonomic assignment of genes associated with ectoine biosynthesis and degradation indicated contributions from both archaeal and bacterial lineages, with clear differences between pathways. Genes involved in ectoine degradation were affiliated with a mixture of *Halobacteriota*, and some representa­tion of *Alphaproteobacteria* and *Gammaproteobacteria*, suggesting both archaeal and bacterial members of the community contribute to osmolyte turnover. In contrast, ectoine biosynthesis genes showed a more uneven taxonomic distribution, with the gene encoding diaminobutyrate-2-oxoglutarate transaminase (*ectB*) predominantly affiliated with Halobacteriota, and some representation of Alpha- and Gammaproteobacteria. However, the downstream pathway genes encoding L‑2,4‑diaminobutyric acid acetyltransferase (*ectA*) and L‑ectoine synthase (*ectC*) were identified exclusively in gammaproteobacterial lineages. This taxonomic pattern indicates that while haloarchaeal taxa dominate the metagenomes and are consistent with a salt‑in strategy based on intracellular potassium accumulation, the genetic potential for ectoine biosynthesis is largely restricted to bacterial members of the community. The coexistence of archaeal‑dominated potassium uptake systems with bacterial-compatible solute production, alongside broadly distributed ectoine degradation genes, supports a model in which organic osmolytes may be synthesised by a subset of *Bacteria* and subsequently utilised, transformed, or recycled by both bacterial and archaeal populations. Ectoine represents one of the most broadly distributed compatible solutes in halophiles, and the selective distribution of ectoine biosynthetic and catabolic gene clusters among halophilic bacterial and archaeal lineages has been linked to salinity-responsive regulation and reutilisation of ectoine as carbon and nitrogen sources [132–135]. Detection of *trkH* alongside genes encoding ectoine biosynthesis and ectoine degradation is consistent with an osmoadaptation strategy in saline and hypersaline systems, in which rapid potassium uptake via Trk/Ktr-type transporters constitutes a primary response to osmotic upshift, while compatible solute pathways underpin longer-term adjustment of cytoplasmic conditions [131, 136, 137]. The co-occurrence of potassium uptake and ectoine cycling genes in the Lake Karum metagenomes therefore indicates genetic potential for a flexible osmoadaptive repertoire.

Genes associated with glycine betaine metabolism were detected at lower relative abundances. Choline and glycine betaine form a tightly linked metabolic continuum in microbial systems, with choline acting as a key precursor for glycine betaine biosynthesis as well as a potential entry point into nitrogen-rich osmolyte turnover [138]. The oxidation of choline to glycine betaine therefore represents not only an osmoadaptive strategy but also a potential mechanism for accessing intracellular pools of reduced nitrogen and methyl groups under saline and nutrient-limited conditions. Enzymes involved in the oxidation of choline to glycine betaine (choline dehydrogenase—*betA* and betaine aldehyde dehydrogenase-*betB*) were present across sediment and water metagenomes. Genes encoding direct methylamine dehydrogenase were absent; however, components of the glutamate-mediated methylamine utilisation pathway (*γ*‑glutamylmethylamide synthetase-*gmaS, N‑*methylglutamate synthase-*mgsC,* and *N*-methylglutamate dehydrogenase-*mgdA*) were detected across samples [139, 140]. The presence of this pathway suggests that methylated nitrogen derived from osmolyte turnover may be assimilated via non‑classical routes, linking compatible solute metabolism with internal nitrogen conservation rather than complete dissimilation. This pathway enables assimilation of methylated nitrogen compounds and may contribute to nitrogen retention under nutrient-limited conditions [141]. The taxonomic profile of these genes was dominated by *Halobacteriota*, suggesting that *Archaea* may contribute to nitrogen retention at the level of genomic potential. Recent advances in the understanding of glycine betaine degradation further indicate that demethylation of this osmolyte can be mechanistically coupled to methionine synthesis and broader nitrogen metabolism, reinforcing the interpretation that osmolyte turnover may play a dual role in osmotic balance and nitrogen recycling in hypersaline systems [142]. Together, these results indicate that osmotic adaptation in Lake Karum is dominated by potassium uptake and ectoine-based strategies, with additional capacity for compatible solute turnover and nitrogen recycling.

### Identification and analysis of metagenome-assembled genomes

Ensemble binning of the Lake Karum metagenomes produced 142 non-redundant MAGs. This includes four MAGs of high quality (completeness score > 95% and contamination < 5%, copies of a complete set of ribosomal genes and > 18 unique tRNAs [143]) and 138 of medium quality (completeness score > 70% and contamination < 10%) [143] (Supplementary Table 7). MAGs of candidate phyla and MAGs with high-quality completion and contamination scores were screened for genes relating to biogeochemical cycling. MAG abundances showed a consistent dominance of *Halobacteriota* in the Lake Karum metagenomes (102 MAGs), with *Halobacteriales* and several halobacterial families and genera present across multiple datasets, although their relative contributions varied between sites (Supplementary Table 8). For example, *Halobaculum* and *Haloarculaceae* MAGs contributed more strongly to the S1-S2 datasets, whereas MAGs resolved only to the *Halobacteriales* level were proportionally more abundant in S3-S4. Non‑halobacterial MAGs (40 MAGs) were consistently detected at lower abundances and showed a patchy distribution across samples; for instance, *Bacteroidota* MAGs were detected predominantly in S3 and S4, while *Pseudomonadota* lineages such as *Limimonas* and *Francisellaceae* were restricted to one or two datasets. Of the *Halobacteriota* MAGs, 56 could only be classified to order or family level. Those Halobacteriota MAGs that could be classified to genus level were *Halobaculum* (13), *Halobellus* (9), *Haloplanus* (4), *Halopricum* (4), *Salinirussus* [4], *Halonotius* (3), *Halobacterium* (2), *Saliniarchaeum* (2), *Halosimplex* (2), *Halorussus* (1), *Haloarcula* (1), and *Halomarina* (1). There was variability in the functional potential of the 102 MAGs identified as *Halobacteriota*. *hhyL* was identified in 24, *coxL* in 65, *narG* in 29, *nirK* in 87, *norB* in 56 and *nosZ* in 23. Therefore, whilst the genes encoding enzymes for hydrogen and carbon monoxide oxidation, and for denitrification, in Lake Karum metagenomes were dominated by *Halobacteriota*, many halobacterial MAGs within Lake Karum either lacked these genes or were only capable of incomplete metabolism. Whilst the lack of detection is not surprising in MAGs with lower completeness, the lack of detection in 10 MAGs with > 90% completeness across six halobacterial lineages indicates that this is potentially a consistent observation. This therefore reinforces the importance of an experimental approach that enables identification of intrageneric variation to better connect microbiome functional and taxonomic diversity. The MAGs classified as taxa other than the *Halobacteriota* were *Candidatus* *Nanohaloarchaeota* (15),* Bacteroidota* (10), *Limimonas* (2), *Candidatus Pacebacteria* (1), *Cyanothece* (1), *Halofilum* (3), *Candidatus* *Bipolaricaulota* (1), *Candidatus Salsurabacteriota* (2), *Dependencia* (2), *Abscondibacteriales* (2), *Francisellaceae* (1) and *Woesarchaeota* (4). The high-quality *Cyanothece* MAG (W5.bin002) was the only MAG to contain a complete *nifDKH* nitrogenase encoding gene operon. The MAG also contained genes encoding a complete photosystem, carbon dioxide fixation pathway, and a hydrogenase (*hhyL*), traits that have been well-characterised in cultivated strains of *Cyanothece* [144–146]. W5.bin002 lacks complete biosynthesis pathways for ectoine, glycine betaine or trehalose. The MAG does contain *kdpA* genes encoding for a potassium transport system, indicating potassium accumulation to maintain osmotic balance. This MAG also possessed a methanethiol *S*-methyltransferase gene (*mddA*), involved in the oxidation of dimethylsulfide [147, 148], which has also been confirmed as metabolically active in cultivated strains of *Cyanothece*. This metabolic potential supports the role of *Cyanothece* in Lake Karum in impacting elemental cycles, including carbon, nitrogen, sulfur, and hydrogen.

Analysis of the other bacterial MAGs identified the presence of additional biogeochemical cycling pathways. The *Halofilum* MAGs contained periplasmic nitrate reductase encoding genes (*nap*), the *Limimonas* MAGs contained genes encoding a nitrate reductase (*narG*) and a carbon monoxide dehydrogenase (*coxL*), and the *Salinibacter* MAGs contained genes encoding nitrite and nitrous oxide reductase (*nirK* and *nosZ*). Therefore, the representatives of abundant bacterial taxa identified via binning only possessed the genetic potential for limited contributions to these elemental cycling pathways (e.g., incomplete denitrification, in line with cultivated representatives of *Salinibacter* and *Halofilum* [149, 150]).

The other MAGs were screened for genes encoding enzymes with potential impact on biogeochemical cycling pathways. Screening of the MAGs assigned to the *Absconditabacterales*, *Candidatus* Pacebacteria or *Candidatus* Nanohaloarchaeota aligned with studies identifying the streamlined nature of the taxa, with a minimal presence of genes relating to central metabolic pathways and biogeochemical cycling [151–156]. The same was observed for the MAGs identified as *Babeliaceae* or *Woesarchaeota,* supporting their identification as syntrophic, predatory, or parasitic [154, 157–160]. However, some MAGs identified as candidate taxa had more extensive metabolic potentials. The *Balneolaceae* and *Phycisphaeraceae* MAGs both possessed genes encoding a nitrous oxide reductase. One high-quality MAG from *Candidatus* Bipolaricaulota RBG-16–55-9 (Supplementary Table 7) possessed genes encoding dissimilatory nitrite reduction to ammonia (*nrfA*), nitrite reduction to nitric oxide (*nirK*), and a carbon monoxide dehydrogenase (*coxL*). Bipolaricaulota MAGs assembled from metagenomes from hypersaline salt flats and marine sediments [27, 41, 161] also possessed these genes, suggesting these metabolic processes are common in this candidate phylum.

Our analysis identified two metagenome-assembled genomes (MAGs) closely related to the bacterial *Candidatus* Salsurabacteriota lineage [44] (also referred to as phylum T1Sed10-126 and *Candidatus* Ceracanota [162–164]), with one identified as high quality (S3.bin.034—96.6% completeness, 1.1% contamination, all rRNAs, and > 18 tRNAs). This MAG is of note as it represents the first MAG of this candidate phylum to possess a full complement of rRNA genes, including the first 16S rRNA gene. The two original MAGs of this taxon were generated from metagenomic sequence data from sediment from a Russian Soda Lake (7 M NaCl and pH 10)[44], with one further MAG from a Polish salt mine [165], and three further MAGs from metagenomes generated from the water of Lake Karum sampled in 2016 and 2020 [59]. Analysis of the 16S rRNA gene sequences from one of the *Candidatus* Salsurabacteriota MAGs generated in this study alongside the ASVs generated from the 16S rRNA gene amplicons, showed that the sequence was present at < 1% relative abundance across the 2018 and 2020 Karum amplicons. As this is the first 16S rRNA gene sequence from a T1SED10-126 MAG, the sequence with the highest identity to the 16S rRNA gene sequence of this MAG in the NCBI database had 93% sequence identity, and this was an environmental sequence from the Dallol salt cave [51, 52], with further hits of > 90% sequence identity identified in Lake Tyrell in Australia [166], the Ebinur Salt Lake in China [167, 168], and saline, intertidal soils in the Gulf of Cambay in India [169], indicating a widespread presence within global saline and hypersaline environments. The MAG generated in this study contained genes for nitric and nitrous oxide reductases, a low-affinity cytochrome C oxidase, and a sulfate adenylyltransferase, consistent with previous findings in Lake Karum [59]. It also possessed genes shared across all currently published *Candidatus* Salsuribacteriota MAGs, including genes involved in flagellar motility, chemotaxis, and bacterial haemoglobin. Bacterial haemoglobin can bind and transport oxygen [170], and could possibly have a role in supporting aerobic respiration in oxygen-poor, hypersaline fluids [171]. This study’s *Candidatus* Salsurabacteriota MAG encodes an F-type ATPase, contrasting with the V-type ATPase found in the original Russian MAGs [44], and methanol dehydrogenase (*xoxF*) absent in previously reported Lake Karum MAGs. Conversely, this study’s *Candidatus* Salsurabacteriota MAG lacks genes encoding sulfate, nitrate, and nitrite reductases, as well as hydrogenases, which were present in the other studies, reinforcing the broad metabolic diversity within this candidate phylum.

## Conclusion

Genome-resolved metagenomic analysis of Lake Karum revealed a hypersaline microbiome dominated by *Halobacteriales*, with bacterial taxa and candidate phyla present in lower abundance. The screening of these MAGs supports the identification of many key genes encoding enzymes involved in biogeochemical cycling and the metabolism of climatically relevant compounds as either Cyanobacteriota or predominantly Halobacteriota within the metagenomic functional profile. The functional and taxonomic diversity identified in the Lake Karum metagenome, and specifically the sequences assigned to the *Halobacteriales* (Oxidation of carbon monoxide, and utilisation of organic sulfur and nitrogen compounds and inorganic nitrogen species), supports *Halobacteriales* having a major impact on the biogeochemical cycling of nitrogen, sulfur and carbon in hypersaline environments [39, 42, 49]. However, analysis of the detected bacterial candidate phyla suggests that some of these taxa may also play a key, as-yet-undefined role in the metabolism of trace gases of atmospheric relevance. While there are variations among sites in the genomic content of MAGs related to climate-relevant metabolisms, there is no observable correlation between the presence or abundance of these pathways and site chemistry. This lack of correlation is consistent with studies indicating that, in addition to salinity, community structure [18] and other unmeasured environmental variables [16, 17] significantly influence microbial metabolic potential in hypersaline systems.

## Supplementary Information

Below is the link to the electronic supplementary material.

Supplementary Material 1

## Data Availability

All sequence data generated in this study was deposited to Sequence Read Archives (SRA) under project number [PRJNA1371326](https:/www.ncbi.nlm.nih.gov/bioproject/PRJNA1371326).

## References

[1] Paul VG, Mormile MR. A case for the protection of saline and hypersaline environments: a microbiological perspective. FEMS Microbiol Ecol. 2017;93:1–14. 10.1093/femsec/fix091 28903468

[2] Andrei AŞ, Banciu HL, Oren A. 2012. Living with salt: Metabolic and phylogenetic diversity of archaea inhabiting saline ecosystems. FEMS Microbiol Lett. 2012;330(1):1–9. 10.1111/j.1574-6968.2012.02526.x 22339687

[3] Rich VI, Maier RM. Aquatic environments. In: Environmental microbiology: third edition. Elsevier Inc; 2015. p. 111–38. 10.1111/j.1574-6968.2012.02526.x

[4] Baricz A, Chiriac CM, Ștefan AA, Bulzu PA, Levei EA, Cadar O, et al. Spatio-temporal insights into microbiology of the freshwater-to-hypersaline, oxic-hypoxic-euxinic waters of Ursu Lake. Environ Microbiol. 2021;23:3523–40. 31894632 10.1111/1462-2920.14909

[5] Filippidou S, Price A, Spencer-Jones C, Scales A, Macey MC, Franchi F, et al. Diversity of microbial mats in the Makgadikgadi Salt Pans, Botswana. Microorganisms. 2024;12:1–13. 10.3390/microorganisms12010147 PMC10818877 38257974

[6] Cavalazzi B, Barbieri R, Gómez F, Capaccioni B, Olsson-Francis K, Pondrelli M, et al. The Dallol Geothermal Area, Northern Afar (Ethiopia) - an exceptional planetary field analog on Earth. Astrobiology. 2019;19:553–78. 30653331 10.1089/ast.2018.1926 PMC6459281

[7] Moors H, De Craen M, Smolders C, Provoost A, Leys N. 2023. The waterbodies of the halo-volcanic Dallol complex: earth analogs to guide us, where to look for life in the universe. Front Microbiol. 2023;14. 10.3389/fmicb.2023.1134760 PMC10382021 37520359

[8] Mcgenity TJ, Oren A. Hypersaline environments. In: Life at extremes: environments, organisms and strategies for survival. 2012. p. 402–34.

[9] Hallsworth JE, Yakimov MM, Golyshin PN, Gillion JLM, D’Auria G, De Lima AF, et al. Limits of life in MgCl2-containing environments: Chaotropicity defines the window. Environ Microbiol. 2007;9:801–13. 17298378 10.1111/j.1462-2920.2006.01212.x

[10] Martin-Cuadrado AB, Senel E, Martínez-García M, Cifuentes A, Santos F, Almansa C, et al. Prokaryotic and viral community of the sulfate-rich crust from Peñahueca ephemeral lake, an astrobiology analogue. Environ Microbiol. 2019;21:3577–600. 31087616 10.1111/1462-2920.14680

[11] Vavourakis CD, Ghai R, Rodriguez-Valera F, Sorokin DY, Tringe SG, Hugenholtz P, Muyzer G. 2016. Metagenomic insights into the uncultured diversity and physiology of microbes in four hypersaline soda lake brines. Front Microbiol. 2016;7. 10.3389/fmicb.2016.00211 PMC4766312 26941731

[12] Riveros-Iregui DA, Lenters JD, Peake CS, Ong JB, Healey NC, Zlotnik VA. Evaporation from a shallow, saline lake in the Nebraska Sandhills: energy balance drivers of seasonal and interannual variability. J Hydrol (Amst). 2017;553:172–87.

[13] Isaji Y, Kawahata H, Ogawa NO, Kuroda J, Yoshimura T, Jiménez-Espejo FJ, et al. Efficient recycling of nutrients in modern and past hypersaline environments. Sci Rep. 2019;9:1–12. 30842491 10.1038/s41598-019-40174-9 PMC6403304

[14] Gu H, Zhang W, Gillies R. The shrinking great salt lake may exacerbate droughts by reducing local precipitation: a case study 10.1175/JHM-D-23.

[15] Wurtsbaugh WA, Miller C, Null SE, Justin De Rose R, Wilcock P, Hahnenberger M, et al. Decline of the world’s saline lakes. Nat Geosci. 2017;10:816–21.

[16] Keneally C, Gaget V, Chilton D, Kidd SP, Mosley L, Welsh DT, et al. Microbial ecology in hypersaline coastal lagoons: A model for climate-induced coastal salinisation and eutrophication. Earth Sci Rev. 2025. 10.1016/j.earscirev.2025.105150.

[17] Morant D, Rochera C, Picazo A, Miralles-Lorenzo J, Camacho-Santamans A, Camacho A. Ecological status and type of alteration determine the C-balance and climate change mitigation capacity of Mediterranean inland saline shallow lakes. Sci Rep. 2024. 10.1038/s41598-024-79578-7. 39580551 PMC11585656

[18] Liu YH, Gao L, Jiang HC, Fang BZ, Huang Y, Li L, et al. Response of microbial diversity and function to the degradation of Barkol Saline Lake. Front Microbiol. 2024. 10.3389/fmicb.2024.1358222. 38784797 PMC11111964

[19] Cobo M, Goldhammer T, Brothers S. A desiccating saline lake bed is a significant source of anthropogenic greenhouse gas emissions. One Earth. 2024;7:1414–23.

[20] Jiang X, Liu C, Hu Y, Shao K, Tang X, Zhang L, et al. Climate-induced salinization may lead to increased lake nitrogen retention. Water Res. 2023. 10.1016/j.watres.2022.119354. 36435160

[21] Williams WD. Environmental threats to salt lakes and the likely status of inland saline ecosystems in 2025. Environ Conserv. 2002;29:154–67.

[22] Rinnan R, Steinke M, Mcgenity T, Loreto F. Plant volatiles in extreme terrestrial and marine environments. Plant Cell Environ. 2014;37:1776–89. 24601952 10.1111/pce.12320

[23] Shadrin NV, Anufriieva EV. Climate change impact on the marine lakes and their crustaceans: the case of marine hypersaline lake Bakalskoye (Ukraine). Turk J Fish Aquat Sci. 2013;13:603–11.

[24] Keneally C, Southgate M, Chilton D, Gaget V, Welsh DT, Mosley L, et al. Organic matter accumulation drives methylotrophic methanogenesis and microbial ecology in a hypersaline coastal lagoon. Limnol Oceanogr. 2024;69:1970–83.

[25] Saccò M, White NE, Harrod C, Salazar G, Aguilar P, Cubillos CF, et al. Salt to conserve: a review on the ecology and preservation of hypersaline ecosystems. Biol Rev. 2021;96:2828–50. 34747117 10.1111/brv.12780

[26] Ricci F, Leung PM, Hutchinson T, Nguyen-Dinh T, Frank AH, Hood AvanS, et al. Chemosynthesis enhances net primary production and nutrient cycling in a hypersaline microbial mat. ISME J. 2025. 10.1093/ismejo/wraf117. 40488405 PMC12218205

[27] McGonigle JM, Bernau JA, Bowen BB, Brazelton WJ. Metabolic potential of microbial communities in the hypersaline sediments of the Bonneville Salt Flats. mSystems. 2022. 10.1128/msystems.00846-22. 36377900 PMC9765009

[28] Valdes DS, Real E. 2004. Nitrogen and phosphorus in water and sediments at Ria Lagartos coastal lagoon, Yucatan, Gulf of MexicoIndian J Mar Sci. 2004.

[29] Foti M, Sorokin DY, Lomans B, Mussman M, Zacharova EE, Pimenov NV, et al. Diversity, activity, and abundance of sulfate-reducing bacteria in saline and hypersaline soda lakes. Appl Environ Microbiol. 2007;73:2093–100. 17308191 10.1128/AEM.02622-06 PMC1855663

[30] King GM. Carbon monoxide as a metabolic energy source for extremely halophilic microbes: implications for microbial activity in Mars regolith. Proc Natl Acad Sci U S A. 2015;112:4465–70. 25831529 10.1073/pnas.1424989112 PMC4394273

[31] Torregrosa-Crespo J, Miralles-Robledillo JM, Bernabeu E, Pire C, Martínez-Espinosa RM. Denitrification in hypersaline and coastal environments. FEMS Microbiol Lett. 2023. 10.1093/femsle/fnad066. 37422443 PMC10423024

[32] Abed RMM, Lam P, De Beer D, Stief P. High rates of denitrification and nitrous oxide emission in arid biological soil crusts from the Sultanate of Oman. ISME J. 2013;7:1862–75. 23575368 10.1038/ismej.2013.55 PMC3749496

[33] Shapovalova AA, Khijniak TV, Tourova TP, Muyzer G, Sorokin DY. Heterotrophic denitrification at extremely high salt and pH by haloalkaliphilic Gammaproteobacteria from hypersaline soda lakes. Extremophiles. 2008;12:619–25. 18452025 10.1007/s00792-008-0166-6 PMC2525850

[34] Magnuson E, Altshuler I, Fernández-Martínez M, Chen YJ, Maggiori C, Goordial J, et al. Active lithoautotrophic and methane-oxidizing microbial community in an anoxic, sub-zero, and hypersaline High Arctic spring. ISME J. 2022;16:1798–808. 35396347 10.1038/s41396-022-01233-8 PMC9213412

[35] Maignien L, Parkes RJ, Cragg B, Niemann H, Knittel K, Coulon S, et al. Anaerobic oxidation of methane in hypersaline cold seep sediments. FEMS Microbiol Ecol. 2013;83:214–31. 22882187 10.1111/j.1574-6941.2012.01466.x

[36] Lay CY, Mykytczuk NCS, Yergeau É, Lamarche-Gagnon G, Greer CW, Whyte LG. Defining the functional potential and active community members of a sediment microbial community in a high-arctic hypersaline subzero spring. Appl Environ Microbiol. 2013;79:3637–48. 23563939 10.1128/AEM.00153-13 PMC3675919

[37] Antony CP, Kumaresan D, Ferrando L, Boden R, Moussard H, Scavino AF, et al. Active methylotrophs in the sediments of Lonar Lake, a saline and alkaline ecosystem formed by meteor impact. ISME J. 2010;4:1470–80. 20555363 10.1038/ismej.2010.70

[38] Dinsdale EA, Edwards RA, Hall D, Angly F, Breitbart M, Brulc JM, et al. Functional metagenomic profiling of nine biomes. Nature. 2008;452:629–32. 18337718 10.1038/nature06810

[39] Sorokin DY, Merkel AY, Messina E, Tugui C, Pabst M, Golyshin PN, et al. Anaerobic carboxydotrophy in sulfur-respiring haloarchaea from hypersaline lakes. ISME J. 2022;16:1534–46. 35132120 10.1038/s41396-022-01206-x PMC9123189

[40] Yau S, Lauro FM, Williams TJ, Demaere MZ, Brown MV, Rich J, et al. Metagenomic insights into strategies of carbon conservation and unusual sulfur biogeochemistry in a hypersaline Antarctic lake. ISME J. 2013;7:1944–61. 23619305 10.1038/ismej.2013.69 PMC3965305

[41] Hazzouri KM, Sudalaimuthuasari N, Saeed EE, Kundu B, Al-Maskari RS, Nelson D, et al. Salt flat microbial diversity and dynamics across salinity gradient. Sci Rep. 2022. 10.1038/s41598-022-15347-8. 35788147 PMC9253026

[42] Miralles-Robledillo JM, Bernabeu E, Giani M, Martínez-Serna E, Martínez-Espinosa RM, Pire C. Distribution of denitrification among haloarchaea: a comprehensive study. Microorganisms. 2021;9:1669. 34442748 10.3390/microorganisms9081669 PMC8400030

[43] Wong HL, MacLeod FI, White RA, Visscher PT, Burns BP. Microbial dark matter filling the niche in hypersaline microbial mats. Microbiome. 2020;8:1–14. 32938503 10.1186/s40168-020-00910-0 PMC7495880

[44] Vavourakis CD, Andrei AS, Mehrshad M, Ghai R, Sorokin DY, Muyzer G. A metagenomics roadmap to the uncultured genome diversity in hypersaline soda lake sediments 06 biological sciences 0605 microbiology 06 biological sciences 0604 genetics. Microbiome. 2018;6:1–18. 30231921 10.1186/s40168-018-0548-7 PMC6146748

[45] Corti G. Continental rift evolution: from rift initiation to incipient break-up in the Main Ethiopian Rift, East Africa. Earth Sci Rev. 2009;96:1–53.

[46] Rime V, Negga H, Fentimen R, Rüggeberg A, El Korh A, Pirkenseer C, et al. Nature and significance of Late Pleistocene to Holocene thick evaporite deposits of the Danakil Depression, Afar, Ethiopia. Sedimentology. 2025;72:475–506.

[47] Jaramillo-Vogel D, Foubert A, Braga JC, Schaegis JC, Atnafu B, Grobety B, et al. Pleistocene sea-floor fibrous crusts and spherulites in the Danakil Depression (Afar, Ethiopia). Sedimentology. 2019;66:480–512.

[48] Ghai R, Pašić L, Fernández AB, Martin-Cuadrado AB, Mizuno CM, McMahon KD, et al. New abundant microbial groups in aquatic hypersaline environments. Sci Rep. 2011;1:135. 22355652 10.1038/srep00135 PMC3216616

[49] Sorokin DiY, Messina E, Smedile F, Roman P, Damsté JSS, Ciordia S, et al. Discovery of anaerobic lithoheterotrophic haloarchaea, ubiquitous in hypersaline habitats. ISME J. 2017;11:1245–60. 28106880 10.1038/ismej.2016.203 PMC5437934

[50] Lay CY, Mykytczuk NCS, Niederberger TD, Martineau C, Greer CW, Whyte LG. Microbial diversity and activity in hypersaline high Arctic spring channels. Extremophiles. 2012;16:177–91. 22246205 10.1007/s00792-011-0417-9

[51] Belilla J, Iniesto M, Moreira D, Benzerara K, López-García JM, López-Archilla AI, et al. Archaeal overdominance close to life-limiting conditions in geothermally influenced hypersaline lakes at the Danakil Depression, Ethiopia. Environ Microbiol. 2021;23:7168–82. 34519149 10.1111/1462-2920.15771

[52] Belilla J, Moreira D, Jardillier L, Reboul G, Benzerara K, López-García JM, et al. Hyperdiverse archaea near life limits at the polyextreme geothermal Dallol area. Nat Ecol Evol. 2019;3:1552–61. 31666740 10.1038/s41559-019-1005-0 PMC6837875

[53] Philippot L, Andersson SGE, Battin TJ, Prosser JI, Schimel JP, Whitman WB, et al. The ecological coherence of high bacterial taxonomic ranks. Nat Rev Microbiol. 2010;8:523–9. 20531276 10.1038/nrmicro2367

[54] Galisteo C, de la Haba RR, Sánchez-Porro C, Ventosa A. A step into the rare biosphere: genomic features of the new genus *Terrihalobacillus* and the new species *Aquibacillus salsiterrae* from hypersaline soils. Front Microbiol. 2023. 10.3389/fmicb.2023.1192059. 37228371 PMC10203224

[55] González-Torres P, Gabaldón T. Genome variation in the model halophilic bacterium *Salinibacter ruber*. Front Microbiol. 2018. 10.3389/fmicb.2018.01499. 30072959 PMC6060240

[56] Viver T, Conrad RE, Orellana LH, Urdiain M, González-Pastor JE, Hatt JK, et al. Distinct ecotypes within a natural haloarchaeal population enable adaptation to changing environmental conditions without causing population sweeps. ISME J. 2021;15:1178–91. 33342997 10.1038/s41396-020-00842-5 PMC8182817

[57] Peña A, Valens M, Santos F, Buczolits S, Antón J, Kämpfer P, et al. Intraspecific comparative analysis of the species *Salinibacter ruber*. Extremophiles. 2005;9:151–61. 15841344 10.1007/s00792-005-0430-y

[58] Martínez-Alvarez L, Ramond JB, Vikram S, León-Sobrino C, Maggs-Kölling G, Cowan DA. With a pinch of salt: metagenomic insights into Namib Desert salt pan microbial mats and halites reveal functionally adapted and competitive communities. Appl Environ Microbiol. 2023. 10.1128/aem.00629-23. 37971255 PMC10734447

[59] Gutiérrez-Preciado A, Dede B, Baker BA, Eme L, Moreira D, López-García P. Extremely acidic proteomes and metabolic flexibility in bacteria and highly diversified archaea thriving in geothermal chaotropic brines. Nat Ecol Evol. 2024. 10.1038/s41559-024-02505-6. 39134651

[60] Macey MC, Fox-Powell M, Ramkissoon NK, Stephens BP, Barton T, Schwenzer SP, et al. The identification of sulfide oxidation as a potential metabolism driving primary production on late Noachian Mars. Sci Rep. 2020. 10.1038/s41598-020-67815-8. 32616785 PMC7331718

[61] Metsalu T, Vilo J. ClustVis: a web tool for visualizing clustering of multivariate data using Principal Component Analysis and heatmap. Nucleic Acids Res. 2015;43:W566–70. 25969447 10.1093/nar/gkv468 PMC4489295

[62] Tillett D, Neilan BA. Xanthogenate nucleic acid isolation from cultured and environmental cyanobacteria. J Phycol. 2000;36:251–8.

[63] Green MR, Sambrook J. Precipitation of DNA with ethanol. Cold Spring Harb Protoc. 2016;2016:1116–20. 10.1101/pdb.prot093377 27934690

[64] Schwieger F, Tebbe CC. A new approach to utilize PCR-single-strand-conformation polymorphism for 16S rRNA gene-based microbial community analysis. Appl Environ Microbiol. 1998;64:4870–6. 9835576 10.1128/aem.64.12.4870-4876.1998 PMC90936

[65] Stach JEM, Bathe S, Clapp JP, Burns RG. PCR-SSCP comparison of 16S rDNA sequence diversity in soil DNA obtained using different isolation and purification methods. FEMS Microbiol Ecol. 2006;36:139–51. 10.1111/j.1574-6941.2001.tb00834.x 11451518

[66] Callahan BJ, McMurdie PJ, Rosen MJ, Han AW, Johnson AJA, Holmes SP. DADA2: high-resolution sample inference from Illumina amplicon data. Nat Methods. 2016;13(7):581-583 10.1038/nmeth.3869. Epub 2016 May 23. PMID: 27214047; PMCID: PMC4927377. PMC4927377 27214047

[67] Quast C, Pruesse E, Yilmaz P, Gerken J, Schweer T, Yarza P, Peplies J, Glöckner FO. The SILVA ribosomal RNA gene database project: improved data processing and web-based tools. Nucleic Acids Res. 2013 Jan;41(Database issue):D590-6. 10.1093/nar/gks1219 PMC3531112 23193283

[68] Wright ES. Using DECIPHER v2.0 to Analyze Big Biological Sequence Data in R. 2016. 10.32614/RJ-2016-025

[69] Schliep KP. Phangorn: phylogenetic analysis in R. Bioinformatics. 2011;27:592–3. 21169378 10.1093/bioinformatics/btq706 PMC3035803

[70] McMurdie PJ, Holmes S. Phyloseq: an R package for reproducible interactive analysis and graphics of microbiome census data. PLoS ONE. 2013. 10.1371/journal.pone.0061217. 23630581 PMC3632530

[71] Oksanen J, Blanchet FG, Kindt R, Legendre P, Minchin PR, O’Hara RB, SimpsonGL, Solymos P, Stevens MHH, Wagner H. Vegan: Community EcologyPackage. R Package Version 2.2–0. 2014.

[72] R Core Team. R: A language and environment for statistical computing. 2016. R Foundation for Statistical Computing, Vienna, Austria.

[73] Oksanen J, Simpson G, Blanchet F, Kindt R, Legendre P, Minchin P, O’Hara R, Solymos P, Stevens M, Szoecs E, Wagner H, Barbour M, Bedward M, Bolker B, Borcard D, Borman T, Carvalho G, Chirico M, De Caceres M, Durand S, Evangelista H, FitzJohn R, Friendly M, Furneaux B, Hannigan G, Hill M, Lahti L, Martino C, McGlinn D, Ouellette M, Ribeiro Cunha E, Smith T, Stier A, Ter Braak C, Weedon J. vegan: Community Ecology Package. R package version 28–0, https://vegandevs.github.io/vegan/.

[74] Wei T, Simko V. corrplot: Visualization of a Correlation Matrix. R packageversion 0.95. 2024. https://github.com/taiyun/corrplot

[75] Bolger AM, Lohse M, Usadel B. Trimmomatic: a flexible trimmer for Illumina sequence data. Bioinformatics. 2014;30:2114–20. 24695404 10.1093/bioinformatics/btu170 PMC4103590

[76] Gurevich A, Saveliev V, Vyahhi N, Tesler G. QUAST: quality assessment tool for genome assemblies. Bioinformatics. 2013;29:1072–5. 23422339 10.1093/bioinformatics/btt086 PMC3624806

[77] Li D, Liu C, Luo R, Sadakane K, Lam T. Sequence analysis MEGAHIT: an ultra-fast single-node solution for large and complex metagenomics assembly via succinct de Bruijn graph. Bioinformatics. 2015;31:1674–6. 25609793 10.1093/bioinformatics/btv033

[78] Seemann T. Prokka: rapid prokaryotic genome annotation. Bioinformatics. 2014;30:2068–9. 24642063 10.1093/bioinformatics/btu153

[79] Menzel P, Ng KL, Krogh A. Fast and sensitive taxonomic classification for metagenomics with Kaiju. Nat Commun. 2016;7:11257. 10.1038/ncomms11257. PMID: 27071849; PMCID: PMC4833860. PMC4833860 27071849

[80] Shaffer M, Borton MA, McGivern BB, Zayed AA, La Rosa SL, Solden LM, et al. DRAM for distilling microbial metabolism to automate the curation of microbiome function. Nucleic Acids Res. 2020;48:8883–900. 32766782 10.1093/nar/gkaa621 PMC7498326

[81] Arkin AP, Cottingham RW, Henry CS, Harris NL, Stevens RL, Maslov S, et al. KBase: the United States department of energy systems biology knowledgebase. Nat Biotechnol. 2018;36:566–9. 29979655 10.1038/nbt.4163 PMC6870991

[82] Kanehisa M, Sato Y, Morishima K. BlastKOALA and GhostKOALA: KEGG tools for functional characterization of genome and metagenome sequences. J Mol Biol. 2016;428:726–31. 26585406 10.1016/j.jmb.2015.11.006

[83] Bateman A, Martin MJ, O’Donovan C, Magrane M, Alpi E, Antunes R, et al. UniProt: The universal protein knowledgebase. Nucleic Acids Res. 2017;45:D158–69. 27899622 10.1093/nar/gkw1099 PMC5210571

[84] Bateman A, Martin MJ, Orchard S, Magrane M, Ahmad S, Alpi E, et al. UniProt: the universal protein knowledgebase in 2023. Nucleic Acids Res. 2023;51:D523–31. 36408920 10.1093/nar/gkac1052 PMC9825514

[85] Wu YW, Simmons BA, Singer SW. MaxBin 2.0: an automated binning algorithm to recover genomes from multiple metagenomic datasets. Bioinformatics. 2016;32(4):605–7. 10.1093/bioinformatics/btv638. Epub 2015 Oct 29. PMID: 26515820. 26515820

[86] Kang DD, Li F, Kirton E, Thomas A, Egan R, An H, et al. MetaBAT 2: an adaptive binning algorithm for robust and efficient genome reconstruction from metagenome assemblies. PeerJ. 2019;2019:1–13. 10.7717/peerj.7359 PMC6662567 31388474

[87] Alneberg J, Bjarnason BS, De Bruijn I, Schirmer M, Quick J, Ijaz UZ, et al. Binning metagenomic contigs by coverage and composition. Nat Methods. 2014;11:1144–6. 25218180 10.1038/nmeth.3103

[88] Sieber CMK, Probst AJ, Sharrar A, Thomas BC, Hess M, Tringe SG, et al. Recovery of genomes from metagenomes via a dereplication, aggregation and scoring strategy. Nat Microbiol. 2018;3:836–43. 29807988 10.1038/s41564-018-0171-1 PMC6786971

[89] Olm MR, Brown CT, Brooks B, Banfield JF. DRep: a tool for fast and accurate genomic comparisons that enables improved genome recovery from metagenomes through de-replication. ISME J. 2017;11:2864–8. 28742071 10.1038/ismej.2017.126 PMC5702732

[90] Parks DH, Imelfort M, Skennerton CT, Hugenholtz P, Tyson GW. CheckM: assessing the quality of microbial genomes recovered from isolates, single cells, and metagenomes. Genome Res. 2015;25:1043–55. 25977477 10.1101/gr.186072.114 PMC4484387

[91] Laczny CC, Sternal T, Plugaru V, Gawron P, Atashpendar A, Margossian HH, et al. VizBin - an application for reference-independent visualization and human-augmented binning of metagenomic data. Microbiome. 2015. 10.1186/s40168-014-0066-1. 25621171 PMC4305225

[92] Chaumeil PA, Mussig AJ, Hugenholtz P, Parks DH. GTDB-Tk v2: memory friendly classification with the genome taxonomy database. Bioinformatics. 2022;38:5315–6. 36218463 10.1093/bioinformatics/btac672 PMC9710552

[93] Meier-Kolthoff JP, Göker M. TYGS is an automated high-throughput platform for state-of-the-art genome-based taxonomy. Nat Commun. 2019. 10.1038/s41467-019-10210-3. 31097708 PMC6522516

[94] Hitch TCA, Riedel T, Oren A, Overmann J, Lawley TD, Clavel T. Automated analysis of genomic sequences facilitates high-throughput and comprehensive description of bacteria. ISME Commun. 2021. 10.1038/s43705-021-00017-z. 36732617 PMC9723785

[95] Strunecký O, Ivanova AP, Mareš J. An updated classification of cyanobacterial orders and families based on phylogenomic and polyphasic analysis. J Phycol. 2023. 10.1111/jpy.13304. 36443823

[96] Lefler FW, Berthold DE, Laughinghouse HD. Cyanoseq: a database of cyanobacterial 16S rRNA gene sequences with curated taxonomy. J Phycol. 2023;59:470–80. 37026389 10.1111/jpy.13335

[97] Robinson CK, Wierzchos J, Black C, Crits-Christoph A, Ma B, Ravel J, et al. Microbial diversity and the presence of algae in halite endolithic communities are correlated to atmospheric moisture in the hyper-arid zone of the Atacama Desert. Environ Microbiol. 2015;17:299–315. 24372972 10.1111/1462-2920.12364

[98] The *Prasiolales* (*Chlorophyta*) of atlantic Europe:an assessment based on morphological, molecular, and ecological data,including the characterization of *Rosenvingiella radicans* (Kützing) comb.nov. J Phycol. 2004;40:977–97.

[99] Oren A. Cyanobacteria in hypersaline environments: biodiversity and physiological properties. Biodivers Conserv: Kluwer Academic Publishers; 2015. 10.1007/s10531-015-0882-z.

[100] Van Run J, Steinitz NH. Ecophysiology of the cyanobacterium Dactylococcopsis salina: effect of light intensity, sulphide and temperature. J Gen Microbiol. 1983;129:1849–1856. 10.1099/00221287-129-6-1849

[101] Pérez V, Cortés J, Marchant F, Dorador C, Molina V, Cornejo-d’ottone M, et al. Aquatic thermal reservoirs of microbial life in a remote and extreme high andean hydrothermal system. Microorganisms. 2020. 10.3390/microorganisms8020208. 32028722 PMC7074759

[102] Finstad KM, Probst AJ, Thomas BC, Andersen GL, Demergasso C, Echeverría A,Amundson RG, Banfield JF. Microbial community structure and the persistenceof cyanobacterial populations in salt crusts of the hyperarid atacamadesert from genome-resolved Metagenomics. Front Microbiol. 2017;8. 10.3389/fmicb.2017.01435 PMC5532433 28804480

[103] Rascovan N, Maldonado J, Vazquez MP, Eugenia Farías M. Metagenomic study of red biofilms from Diamante Lake reveals ancient arsenic bioenergetics in haloarchaea. ISME J. 2016;10:299–309. 26140530 10.1038/ismej.2015.109 PMC4737923

[104] Mogany T, Swalaha FM, Allam M, Mtshali PS, Ismail A, Kumari S, et al. Phenotypic and genotypic characterisation of an unique indigenous hypersaline unicellular cyanobacterium, *Euhalothece* sp.nov. Microbiol Res. 2018;211:47–56. 29705205 10.1016/j.micres.2018.04.001

[105] Oren A. Salts and brines. In: Ecology of cyanobacteria II: their diversity in space and time. Springer Netherlands; 2012. p. 401–26.

[106] Walsby AE, Van Rijn J, Cohen Y. The biology of a new gas-vacuolate cyanobacterium, *Dactylococcopsis salina* sp. nov., in Solar Lake. Proc R Soc Lond B Biol Sci. 1983. 10.1098/rspb.1983.0019.

[107] Waditee-Sirisattha R, Ito H, Kageyama H. 2022. Global transcriptional and circadian regulation in a halotolerant cyanobacterium *Halothece* sp. PCC7418. Sci Rep 12. 10.1038/s41598-022-17406-6 PMC9374696 35962002

[108] Fernández-Juárez V, Bennasar-Figueras A, Sureda-Gomila A, Ramis-Munar G,Agawin NSR. Differential effects of varying concentrations of phosphorus,iron, and nitrogen in N_2_-fixing cyanobacteria. Front Microbiol. 2020;11. 10.3389/fmicb.2020.541558 PMC7546424 33101223

[109] Jones CM, Graf DRH, Bru D, Philippot L, Hallin S. The unaccounted yet abundant nitrous oxide-reducing microbial community: a potential nitrous oxide sink. ISME J. 2013;7:417–26. 23151640 10.1038/ismej.2012.125 PMC3554408

[110] Islam ZF, Cordero PRF, Feng J, Chen YJ, Bay SK, Jirapanjawat T, et al. Two Chloroflexi classes independently evolved the ability to persist on atmospheric hydrogen and carbon monoxide. ISME J. 2019;13:1801–13. 30872805 10.1038/s41396-019-0393-0 PMC6776052

[111] Jordaan K, Lappan R, Dong X, Aitkenhead IJ, Bay SK, Chiri E, Wieler N, Meredith LK, Cowan DA, Chown SL, Greening C. Hydrogen-Oxidizing Bacteria Are Abundant in Desert Soils and Strongly Stimulated by Hydration. mSystems. 2020;5(6):e01131-20. 10.1128/mSystems.01131-20.. PMID: 33203691; PMCID: PMC7677003. PMC7677003 33203691

[112] Bay SK, Dong X, Bradley JA, Leung PM, Grinter R, Jirapanjawat T, et al. Trace gas oxidizers are widespread and active members of soil microbial communities. Nat Microbiol. 2021;6:246–56. 33398096 10.1038/s41564-020-00811-w

[113] Adam PS, Borrel G, Gribaldo S. Evolutionary history of carbon monoxide dehydrogenase/acetyl-CoA synthase, one of the oldest enzymatic complexes. Proc Natl Acad Sci U S A. 2018;115:E1166–73. 29358391 10.1073/pnas.1716667115 PMC5819426

[114] Dawson RA, Fantom N, Martin-Pozas T, Aguila P, King GM, Hernández M. Carbon monoxide-oxidising Pseudomonadota on volcanic deposits. Environ Microbiome. 2025;20(1):12. 10.1186/s40793-025-00672-y. PMID: 39865271; PMCID: PMC11771112. PMC11771112 39865271

[115] Meyer O, Frunzke K, Morsdorf G. Biochemistry of the aerobic utilization of carbon monoxide, p. In: Murrell JC, Kelly DP, editors. Microbial growth on C₁ compounds : 7th International symposium : Papers. Intercept; 1993.

[116] Mörsdorf G, Frunzke K, Gadkari D, Meyer O. Microbial growth on carbon monoxide. In: Biodegradation. Kluwer Academic Publishers; 1992.

[117] King GM, Weber CF. Distribution, diversity and ecology of aerobic CO-oxidizing bacteria. Nat Rev Microbiol. 2007. 10.1038/nrmicro1595. 17224920

[118] Zelenski ME, Fischer TP, de Moor JM, Marty B, Zimmermann L, Ayalew D, et al. Trace elements in the gas emissions from the Erta Ale volcano, Afar, Ethiopia. Chem Geol. 2013;357:95–116.

[119] De Moor JM, Fischer TP, Sharp ZD, King PL, Wilke M, Botcharnikov RE, et al. Sulfur degassing at Erta Ale (Ethiopia) and Masaya (Nicaragua) volcanoes: implications for degassing processes and oxygen fugacities of basaltic systems. Geochem Geophys Geosyst. 2013;14:4076–108.

[120] Vavourakis CD, Mehrshad M, Balkema C, van Hall R, Andrei AŞ, Ghai R, Sorokin DY, Muyzer G. Metagenomes and metatranscriptomes shed new light on the microbial-mediated sulfur cycle in a Siberian soda lake. BMC Biol. 2019;17(1):69. 10.1186/s12915-019-0688-7. PMID: 31438955; PMCID: PMC6704655. PMC6704655 31438955

[121] Lee H, Hwang K, Cho A, Kim S, Kim M, Morgan-Kiss R, Priscu JC, Kim KM, Kim OS. Microbial assemblages and associated biogeochemical processes in Lake Bonney, a permanently ice-covered lake in the McMurdo Dry Valleys, Antarctica. Environ Microbiome. 2024;19(1):60. 10.1186/s40793-024-00605-1. PMID: 39160591; PMCID: PMC11334312. PMC11334312 39160591

[122] Liu L, Chen Y, Shen J, Pan Y, Lin W. Metabolic versatility of soil microbial communities below the rocks of the hyperarid Dalangtan Playa. Appl Environ Microbiol. 2023;89(11):e0107223. 10.1128/aem.01072-23PMID: 37902391; PMCID: PMC10686078. PMC10686078 37902391

[123] Sauma-Sánchez T, Alcorta J, Tamayo-Leiva J, Díez B, Bezuidenhout H, Cowan DA, Ramond JB. Functional redundancy buffers the effect of poly-extreme environmental conditions on southern African dryland soil microbial communities. FEMS Microbiol Ecol. 2024;100(12):fiae157. 10.1093/femsec/fiae157. PMID: 39568064; PMCID: PMC11636270. PMC11636270 39568064

[124] Zeng F, Zhu Y, Zhang D, Zhao Z, Li Q, Ma P, Zhang G, Wang Y, Wu S, Guo S, Sun G. Metagenomic analysis of the soil microbial composition and salt tolerance mechanism in Yuncheng Salt Lake, Shanxi Province. Front Microbiol. 2022;13:1004556. 10.3389/fmicb.2022.1004556. PMID: 36225369; PMCID: PMC9549588. PMC9549588 36225369

[125] Yang C, Zhang H, Zhao X, Liu P, Wang L, Wang W. A functional metagenomics study of soil carbon and nitrogen degradation networks and limiting factors on the Tibetan plateau. Front Microbiol. 2023;14:1170806. 10.3389/fmicb.2023.1170806PMID: 37228377; PMCID: PMC10203874. PMC10203874 37228377

[126] Sorokin DY, Yakimov M, Messina E, Merkel AY, Bale NJ, Sinninghe Damste JS. *Natronolimnobius sulfurireducens* sp. nov. and *Halalkaliarchaeum desulfuricum* gen. nov., sp. nov., the first sulfur-respiring alkaliphilic haloarchaea from hypersaline alkaline lakes. Int J Syst Evol Microbiol. 2019;69:2662–73. 31166158 10.1099/ijsem.0.003506

[127] Pol A, Barends TRM, Dietl A, Khadem AF, Eygensteyn J, Jetten MSM, et al. Rare earth metals are essential for methanotrophic life in volcanic mudpots. Environ Microbiol. 2014;16:255–64. 24034209 10.1111/1462-2920.12249

[128] Keltjens JT, Pol A, Reimann J, Op Den Camp HJM. PQQ-dependent methanol dehydrogenases: rare-earth elements make a difference. Appl Microbiol Biotechnol. 2014. 10.1007/s00253-014-5766-8. 24816778

[129] Vu HN, Subuyuj GA, Vijayakumar S, Good NM, Martinez-Gomez NC, Skovran E. Lanthanide-dependent regulation of methanol oxidation systems in *Methylobacterium extorquens* AM1 and their contribution to methanol growth. J Bacteriol. 2016;198:1250–9. 26833413 10.1128/JB.00937-15 PMC4859578

[130] Stautz J, Hellmich Y, Fuss MF, Silberberg JM, Devlin JR, Stockbridge RB, et al. Molecular mechanisms for bacterial potassium homeostasis. J Mol Biol. 2021. 10.1016/j.jmb.2021.166968. 33798529 PMC9041122

[131] Ding R, Yang N, Liu J. The osmoprotectant switch of potassium to compatible solutes in an extremely halophilic archaea *Halorubrum kocurii* 2020YC7. Genes. 2022. 10.3390/genes13060939. PMC9222508 35741701

[132] Liu M, Liu H, Shi M, Jiang M, Li L, Zheng Y. Microbial production of ectoine and hydroxyectoine as high-value chemicals. Microb Cell Fact BioMed. 2021. 10.1186/s12934-021-01567-6. PMC7995798 33771157

[133] Hermann L, Mais CN, Czech L, Smits SHJ, Bange G, Bremer E. The ups and downs of ectoine: structural enzymology of a major microbial stress protectant and versatile nutrient. Biol Chem. 2020. 10.1515/hsz-2020-0223. 32755967

[134] Boas Lichty KE, Thomas HE, Bhide SM, Richards GP, Boyd EF. Deciphering the evolutionary history of ectoine catabolism, a compatible solute utilized by *Vibrio diabolicus* as an osmoprotectant and a nutrient source. Appl Environ Microbiol. 2026. 10.1128/aem.00409-26. 42007718 PMC13188858

[135] Gregory GJ, Boyd EF. Stressed out: Bacterial response to high salinity using compatible solute biosynthesis and uptake systems, lessons from Vibrionaceae. Comput Struct Biotechnol J. 2021. 10.1016/j.csbj.2021.01.030. 33613867 PMC7876524

[136] Oren A. Thermodynamic limits to microbial life at high salt concentrations. Environmental microbiology. 2011;13:1908–23. 10.1111/j.1462-2920.2010.02365.x 21054738

[137] Bremer E, Krämer R. Responses of microorganisms to osmotic stress. Annu Rev Microbiol. 2019;73:313–34. 31180805 10.1146/annurev-micro-020518-115504

[138] Lidbury I, Kimberley G, Scanlan DJ, Murrell JC, Chen Y. Comparative genomics and mutagenesis analyses of choline metabolism in the marine *Roseobacter* clade. Environ Microbiol. 2015;17:5048–62. 26058574 10.1111/1462-2920.12943 PMC4744692

[139] Latypova E, Yang S, Wang YS, Wang T, Chavkin TA, Hackett M, et al. Genetics of the glutamate-mediated methylamine utilization pathway in the facultative methylotrophic beta-proteobacterium *Methyloversatilis universalis* FAM5. Mol Microbiol. 2010;75:426–39. 19943898 10.1111/j.1365-2958.2009.06989.x

[140] Chen Y, McAleer KL, Colin Murrell J. Monomethylamine as a nitrogen source for a nonmethylotrophic bacterium, *Agrobacterium tumefaciens*. Appl Environ Microbiol. 2010;76:4102–4. 20400554 10.1128/AEM.00469-10 PMC2893479

[141] Taubert M, Grob C, Howat AM, Burns OJ, Pratscher J, Jehmlich N, et al. Methylamine as a nitrogen source for microorganisms from a coastal marine environment. Environ Microbiol. 2017;19:2246–57. 28244196 10.1111/1462-2920.13709

[142] Mausz MA, Murphy ARJ, Del Mar Aguilo-Ferretjans M, Hitchcock A, Moran MA, Scanlan DJ, Chen Y, Lidbury IDEA. Methionine synthesis and glycine betaine demethylation are intricately intertwined in cosmopolitan marine bacteria. Proc Natl Acad Sci U S A. 2025;122(38):e2426167122. 10.1073/pnas.2426167122. PMID: 40956897; PMCID: PMC12478193. PMC12478193 40956897

[143] Bowers RM, Kyrpides NC, Stepanauskas R, Harmon-Smith M, Doud D, Reddy TBK, et al. Minimum information about a single amplified genome (MISAG) and a metagenome-assembled genome (MIMAG) of bacteria and archaea. Nat Biotechnol: Nature Publishing Group; 2017. 10.1038/nbt.3893. PMC6436528 28787424

[144] Bandyopadhyay A, Elvitigala T, Welsh E, Stöckel J, Liberton M, Min H, Sherman LA, Pakrasi HB. 2011. Novel metabolic attributes of the genus Cyanothece, comprising a group of unicellular nitrogen-fixing cyanobacteria. mBio 2. 10.1128/mBio.00214-11 PMC3187577 21972240

[145] Colon-Lopez MS, Tang H-Y, Tucker DL, Sherman LA. Analysis of the *nifHDK* operon and structure of the NifH protein from the unicellular, diazotrophic cyanobacterium, *Cyanothece* strain sp. ATCC 511421. Biochim Biophys Acta. 1999;1473:363–75. 10594374 10.1016/s0304-4165(99)00196-8

[146] Wilson ST, Tozzi S, Foster RA, Ilikchyan I, Kolber ZS, Zehr JP, Karl DM. Hydrogen cycling by the unicellular marine diazotroph Crocosphaera watsonii strain WH8501. Appl Environ Microbiol. 2010;76(20):6797–803. 10.1128/AEM.01202-10. PMID: 20709832; PMCID: PMC2953037. PMC2953037 20709832

[147] Carrión O, Pratscher J, Curson ARJ, Williams BT, Rostant WG, Colin Murrell J, et al. Methanethiol-dependent dimethylsulfide production in soil environments. ISME J. 2017;11:2379–90. 28763056 10.1038/ismej.2017.105 PMC5607357

[148] Carrión O, Curson ARJ, Kumaresan D, Fu Y, Lang AS, Mercadé E, Todd JD. A novel pathway producing dimethylsulphide in bacteria is widespread in soil environments. Nat Commun. 2015;6:6579. 10.1038/ncomms7579. PMID: 25807229. 25807229

[149] Xia J, Zhao JX, Sang J, Chen GJ, Du ZJ. *Halofilum ochraceum* gen. Nov. sp.Nov. a gammaproteobacterium isolated from a marine solar saltern. Int J SystEvol Microbiol. 2017. 10.1099/ijsem.0.001718. 27930270

[150] Bagheri M, Marashi SA, Amoozegar MA. A genome-scale metabolic network reconstruction of extremely halophilic bacterium *Salinibacter ruber*. PLoS ONE. 2019. 10.1371/journal.pone.0216336. 31071110 PMC6508672

[151] Tian R, Ning D, He Z, Zhang P, Spencer SJ, Gao S, Shi W, Wu L, Zhang Y, Yang Y, Adams BG, Rocha AM, Detienne BL, Lowe KA, Joyner DC, Klingeman DM, Arkin AP, Fields MW, Hazen TC, Stahl DA, Alm EJ, Zhou J. Small and mighty: adaptation of superphylum Patescibacteria to groundwater environment drives their genome simplicity. Microbiome. 2020;8(1):51. 10.1186/s40168-020-00825-w. PMID: 32252814; PMCID: PMC7137472. PMC7137472 32252814

[152] Lemos LN, Medeiros JD, Dini-Andreote F, Fernandes GR, Varani AM, Oliveira G, et al. Genomic signatures and co-occurrence patterns of the ultra-small Saccharimonadia (phylum CPR/Patescibacteria) suggest a symbiotic lifestyle. Mol Ecol. 2019;28:4259–71. 31446647 10.1111/mec.15208

[153] Narasingarao P, Podell S, Ugalde JA, Brochier-Armanet C, Emerson JB, Brocks JJ, et al. De novo metagenomic assembly reveals abundant novel major lineage of Archaea in hypersaline microbial communities. ISME J. 2012;6:81–93. 21716304 10.1038/ismej.2011.78 PMC3246234

[154] Vigneron A, Cruaud P, Lovejoy C, Vincent WF. Genomic evidence of functional diversity in DPANN archaea, from oxic species to anoxic vampiristic consortia. ISME Commun. 2022;2(1):4. 10.1038/s43705-022-00088-6. PMID: 37938653; PMCID: PMC9723730. PMC9723730 37938653

[155] Wang Y, Gallagher LA, Andrade PA, Liu A, Humphreys IR, Turkarslan S, et al. Genetic manipulation of Patescibacteria provides mechanistic insights into microbial dark matter and the epibiotic lifestyle. Cell. 2023;186:4803-4817.e13. 37683634 10.1016/j.cell.2023.08.017 PMC10633639

[156] Moreira D, Zivanovic Y, López-Archilla AI, Iniesto M, López-García P. Reductive evolution and unique predatory mode in the CPR bacterium Vampirococcus lugosii. Nat Commun. 2021;12(1):2454. 10.1038/s41467-021-22762-4. PMID: 33911080; PMCID: PMC8080830. PMC8080830 33911080

[157] Castelle CJ, Méheust R, Jaffe AL, Seitz K, Gong X, Baker BJ, Banfield JF. Protein Family Content Uncovers Lineage Relationships and Bacterial Pathway Maintenance Mechanisms in DPANN Archaea. Front Microbiol. 2021;12:660052. 10.3389/fmicb.2021.660052. PMID: 34140936; PMCID: PMC8204110. PMC8204110 34140936

[158] Yakimov MM, Merkel AY, Gaisin VA, Pilhofer M, Messina E, Hallsworth JE, et al. Cultivation of a vampire: ‘Candidatus *Absconditicoccus praedator*’. Environ Microbiol. 2022;24:30–49. 34750952 10.1111/1462-2920.15823

[159] Castelle CJ, Wrighton KC, Thomas BC, Hug LA, Brown CT, Wilkins MJ, et al. Genomic expansion of domain archaea highlights roles for organisms from new phyla in anaerobic carbon cycling. Curr Biol. 2015;25:690–701. 25702576 10.1016/j.cub.2015.01.014

[160] Yeoh YK, Sekiguchi Y, Parks DH, Hugenholtz P. Comparative genomics ofcandidate phylum TM6 suggests that parasitism is widespread and ancestralin this lineage. Mol Biol Evol. 2016;33:915–27. 10.1093/molbev/msv281 PMC4776705 26615204

[161] Rogers TJ, Buongiorno J, Jessen GL, Schrenk MO, Fordyce JA, de Moor JM, et al. Chemolithoautotroph distributions across the subsurface of a convergent margin. ISME J. 2023;17:140–50. 36257972 10.1038/s41396-022-01331-7 PMC9751116

[162] Pallen MJ, Rodriguez-R LM, Alikhan NF. Naming the unnamed: over 65,000 Candidatus names for unnamed Archaea and Bacteria in the Genome Taxonomy Database. Int J Syst Evol Microbiol. 2022 Sep;72(9). 10.1099/ijsem.0.005482. PMID: 36125864. 36125864

[163] Ciulla RA, Diaz MR, Taylor BF, Roberts MF. Organic osmolytes in aerobic Bacteria from Mono Lake, an alkaline, moderately hypersaline environment. Appl Environ Microbiol. 1997. 10.1128/aem.63.1.220-226.1997. 16535487 PMC1389101

[164] Zhang XH, Liu J, Liu J, Yang G, Xue CX, Curson ARJ, et al. Biogenic production of DMSP and its degradation to DMS—their roles in the global sulfur cycle. Sci China Life Sci: Science in China Press; 2019. 10.1007/s11427-018-9524-y. 31231779

[165] Lach J, Królikowska K, Baranowska M, Krupińska M, Strapagiel D, Matera-Witkiewicz A, et al. A first insight into the Polish Bochnia Salt Mine metagenome. Environ Sci Pollut Res. 2023;30:49551–66. 10.1007/s11356-023-25770-7 PMC10104926 36780083

[166] Podell S, Ugalde JA, Narasingarao P, Banfield JF, Heidelberg KB, Allen EE. Assembly-driven community genomics of a hypersaline microbial ecosystem. PLoS ONE. 2013. 10.1371/journal.pone.0061692. 23637883 PMC3630111

[167] He Y, Hu W, Ma D, Lan H, Yang Y, Gao Y. Abundance and diversity of ammonia-oxidizing archaea and bacteria in the rhizosphere soil of three plants in the Ebinur Lake wetland. Can J Microbiol. 2017. 10.1139/cjm-2016-0492. 28249125

[168] Zhao S, Liu JJ, Banerjee S, Zhou N, Zhao ZY, Zhang K, Tian CY. Soil pH is equally important as salinity in shaping bacterial communities in saline soils under halophytic vegetation. Sci Rep. 2018;8(1):4550. 10.1038/s41598-018-22788-7PMID: 29540760; PMCID: PMC5851986. PMC5851986 29540760

[169] Keshri J, Yousuf B, Mishra A, Jha B. The abundance of functional genes, *cbbL*, *nifH*, *amoA* and *apsA*, and bacterial community structure of intertidal soil from Arabian Sea. Microbiol Res. 2015;175:57–66. 25862282 10.1016/j.micres.2015.02.007

[170] Frey AD, Kallio PT. Bacterial hemoglobins and flavohemoglobins: versatile proteins and their impact on microbiology and biotechnology. FEMS Microbiol Rev. 2003;27(4):525–45. 10.1016/S0168-6445(03)00056-1. PMID: 14550944. 14550944

[171] Sherwood JE, Stagnitti F, Kokkinn MJ, Williams WD. Dissolved oxygen concentrations in hypersaline waters. Limnol Oceanogr. 1991;36:235–50.

